# The Temperature Dependence of the Pathogenic Bacterial Adhesin Complex SdrG:Fgβ under Sustained Loads: A High-Force Slip Bond

**DOI:** 10.1021/acsnano.6c10566

**Published:** 2026-10-23

**Authors:** Brandon Dzuba, Surya Pratap S. Deopa, Marc-André LeBlanc, Lukas F. Milles, Thomas T. Perkins

**Affiliations:** † JILA, National Institute of Standards and Technology and University of Colorado, Boulder, Colorado 80309, United States; ‡ Gene Center, LMU Munich, Feodor-Lynen-Str. 25, 81377 Munich, Germany; § Max Planck Institute of Biochemistry, Am Klopferspitz 18, 82152 Martinsried, Germany; ∥ Department of Molecular, Cellular, and Developmental Biology, University of Colorado, Boulder, Colorado 80309, United States

**Keywords:** atomic force microscopy, single-molecule force spectroscopy, force clamp, bacterial adhesins, thermodynamics, SdrG, SdrE

## Abstract

*Staphylococcus epidermidis* is the leading cause of medical-implant-related infections. This pathogen attaches to medical implants coated in host fibrinogen via adhesin proteins. To resist high shear forces, adhesin-ligand rupture forces can exceed 2000 pN, a force that is much higher than streptavidin–biotin (150–300 pN). Yet, these adhesin-ligand complexes have a quite moderate *K*_D_ (∼380 nM). This nanomechanics is explained, in part, by catch-bond behavior where adhesin-ligand lifetimes increase with force. Notably, prior reports of adhesin catch-bond mechanics at both low (3–30 pN) and high (1100–1400 pN) forces have lifetimes that are difficult to reconcile. Here, we combined constant-velocity and constant-force assays and thereby showed slip-bond behavior over six decades of lifetimes first for SdrG:Fgβ (1000–2400 pN) and then for SdrE:CFH (800–2200 pN) from *Staphylococcus aureus*. Individual lifetimes for SdrE:CFH exceeded 15 min at 800 pN. To investigate the thermodynamics of SdrG:Fgβ stability, we measured its lifetime using the constant-force assay as a function of both force (1000–1800 pN) and temperature (10–37 °C). Analysis yielded the enthalpic-dissociation barrier (Δ*H* ^‡^ = 40 ± 4 *k*_B_ *T*) and, assuming an enthalpy-dominated barrier, the dissociation attempt frequency (∼6 × 10^10^ s^–^^1^). Our results unambiguously demonstrate high-force slip-bond behavior for two different adhesin-ligand pairs and provide biophysical parameters governing the stability of key virulence factors.

## Graphical abstract

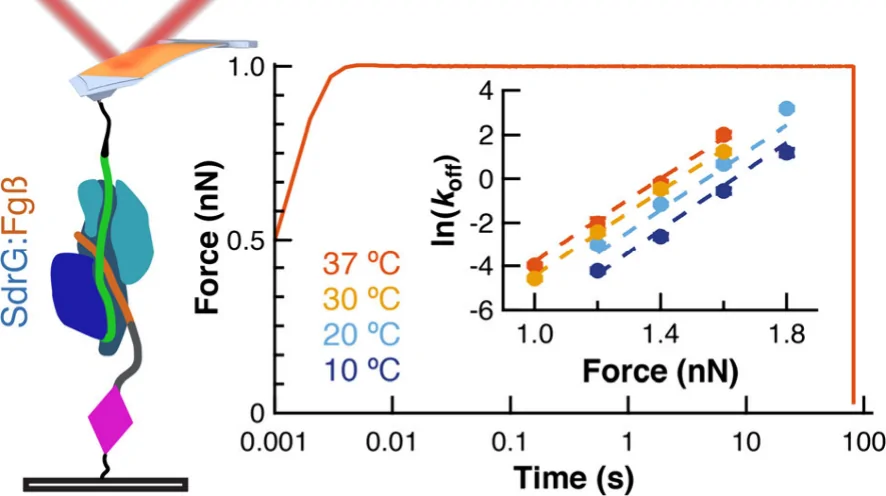

*Staphylococcus epidermidis* is the leading cause of infections on biomedical implants.^1,2^ Implanted medical devices, such as urinary catheters and mechanical heart valves, are rapidly coated by fibrinogen as part of the immune response.^3^ Gram-positive bacteria, like *S. epidermidis*, use bacterial adhesins to bind to this matrix^4^ (Figure 1A), resulting in antibiotic-resistant biofilm formation and chronic infection.^5^ *S. epidermidis* specifically uses the N2 and N3 domains of the SD-repeat-containing protein G (SdrG) to bind a short N-terminal segment [15 amino acid (aa)] of the fibrinogen β chain (Fgβ) (Figure 1B). This SdrG:Fgβ complex achieves extreme mechanical stability via a “dock, lock, and latch” mechanism^6^ that buries the Fgβ peptide in a rigid, screw-like binding pocket, requiring the cooperative rupture of a dense network of backbone hydrogen bonds.^7^ Understanding how this interaction—key for biofilm formation—behaves under sustained high loads and at physiological temperatures is critical to developing a more detailed picture of biofilm formation.

**Figure 1:**
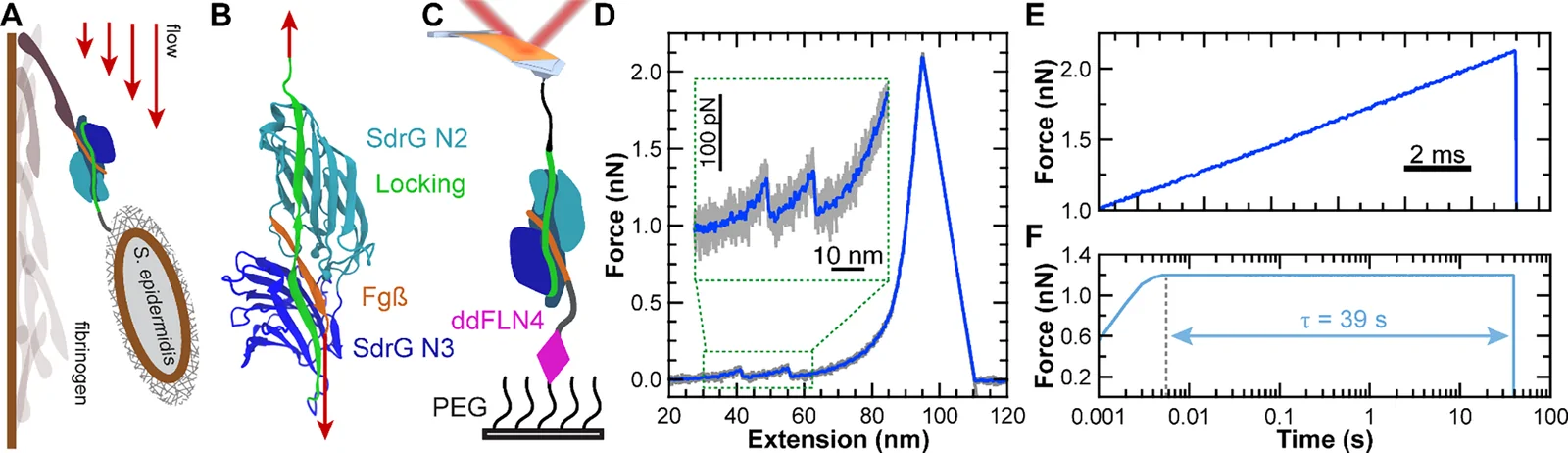
Constant-velocity and constant-force assays for measuring the rupture forces and lifetimes of adhesin complexes. (**A**) Cartoon representation of *Staphylococcus epidermidis* using the N2 and N3 domains of SdrG (cyan and blue, respectively) to bind to Fgβ, a 15-aa segment of fibrinogen (orange), enabling it to resist detachment by shear forces (red arrows). Adapted from ref 7. (**B**) A ribbon diagram illustrates the “dock, lock, and latch” mechanism, whereby Fgβ forms a β-sheet buried between the N2 and N3 domains. The locking strand (light green) locks Fgβ into its binding pocket. Red arrows show the biologically relevant direction of force application. (**C**) Schematic shows the single-molecule assay that mimics this force application. A polypeptide composed of Fgβ and ddFLN4, a marker protein, is covalently coupled to a PEG-coated glass coverslip. SdrG is covalently coupled to a PEG-coated AFM cantilever. (**D**) A force-extension curve measured by retracting the cantilever at a fixed velocity (*v* = 300 nm/s). The characteristic double-peaked unfolding signature of ddFLN4 (inset) is used to verify single-molecule attachment. (**E**) A force-versus-time trace for the record shown in panel D illustrates high forces are applied only very briefly in the constant-velocity assay. (**F**) A force-versus-time trace shows a constant force (1200 pN) applied until the ligand dissociates after a time τ.

Atomic-force-microscopy (AFM) based force-spectroscopy studies of SdrG:Fgβ show interaction rupture forces exceeding 2000 pN, approaching that of covalent bonds,^8^ first using a single cell-based,^9^ and then in an *in vitro* single-molecule assays.^7^ In the *in vitro* single-molecule studies, an SdrG-coated AFM cantilever binds to a surface-anchored polyprotein with Fgβ at its N-terminus to mimic the physiologically relevant direction of applied force (Figure 1C). The cantilever is then rapidly retracted at a constant velocity at room temperature to measure the complex rupture force (Figure 1D). Using this assay, one of us previously reported rupture forces >2000 pN and a linear dependence of the average rupture force as a function of log of the loading rate (∂*F*/∂*t*), in agreement with the Bell–Evans model.^10,11^ A fit to the Bell–Evans model yielded two key free-energy landscape parameters: the distance to the transition state (Δ*x* ^‡^ = 0.051 nm) and the zero-force off rate (*k*_0_ = 9 × 10^–11^ s^–1^). This result demonstrates canonical slip-bond behavior (where lifetimes decrease exponentially with force) above 2000 pN.^7^ Nonetheless, a key limitation of this assay is the complex is only subjected to the high forces for a few milliseconds (Figure 1E), whereas adhesin-ligand complexes anchoring bacteria to medical-device surfaces will encounter longer hydrodynamic challenges, including ∼0.3-s pulsatile systolic flow across prosthetic heart valves,^12^ and ∼10–100-s intermittent urinary-catheter drainage events.^13^

Magnetic-tweezer-based single-molecule force spectroscopy excels at applying constant forces over extended times, but probes a much lower force range.^14^ Huang et al. characterized SdrG:Fgβ and several other adhesin-ligand pairs under constant loads at various temperatures.^15^ At 37 °C, they measured a SdrG:Fgβ lifetime of 3.7 s at 2.6 pN that increased exponentially with force to 754 s at 29 pN, revealing catch-bond behavior (where lifetime increases with force). These results also held for related *S. aureus* adhesin complexes SdrE:CFH and Bbp:Fgα. They posited a low-force dissociation pathway—distinct from the high-force pathway described in previous work^7^—where the locking strand (Figure 1B, green) unbinds, allowing the ligand to dissociate laterally from the binding pocket. This viewpoint is reinforced by recent molecular-dynamics (MD) simulations,^16^ which show distinct force-propagation pathways for the high-force and the low-force dissociation of the SdrG:Fgβ interaction. This mechanism provides a potential explanation for the relatively moderate dissociation constant of SdrG:Fgβ (*K*_D_ ≈ 380 nM)^17^ despite its exceptional mechanical stability under high loads.^16^

For the adhesin-ligand pair SdrE:CFH, both single-molecule magnetic-tweezers^15^ (≤10.5 pN) and single-cell AFM-based force-spectroscopy^18^ (1100–1700 pN) assays report catch-bond behavior. However, the lifetimes measured by magnetic tweezers (∼300 s @ 10 pN, 37 °C) are ∼30-fold longer than those reported in the single-cell assay (8.8 s @ 1400 pN, 20 °C), despite their measurements being at higher temperatures and much lower forces. This discrepancy could be explained by a more complicated force-dependent dissociation pathway than the one previously discussed (i.e., a low-force and high-force pathway).^15,16^ There would need to be at least two additional transitions between catch-bond and slip-bond behavior within the 10.5–1100 pN force range in addition to the one reported at 1400 pN for the cell-based assay. Such a complicated force-dependent dissociation pathway seems implausible and, to our knowledge, has not been observed in other systems. Alternatively, unknown bonds along the force-propagation pathway in the single-cell studies could be rupturing before the adhesin-ligand pair, or multiple molecules binding simultaneously to the tip, giving the appearance of high-force catch-bond dynamics for SdrE:CFH. This discrepancy—together with the lack of high-force measurements at physiological temperatures—indicates an incomplete understanding of the force-response of bacterial adhesins and highlights the need for a high-force, constant-load, *in vitro* single-molecule assay with a mechanically robust surface-anchoring scheme.

Here, we first explored the kinetics and thermodynamics of the adhesin-ligand pair SdrG:Fgβ using both constant-velocity and constant-force assays, collecting over 4,400 single-molecule records. Constant-force data—designed to better mimic extended physiological force application (Figure 1F)—was taken over a range of forces (1000–1800 pN) and temperatures (10–37 °C). Average lifetimes exceeded 50 s under a 1000 pN load at 37 °C. We fit the measured off rate (*k*_off_) as a function of both force and temperature and thereby determined Δ*x* ^‡^ [0.046 ± 0.013 nm (fit ± SD)] and the enthalpic dissociation barrier height (Δ*H* ^‡^ = 40 ± 4 *k*_B_ *T*) using a global fit to the Dudko-Hummer-Szabo (DHS) model.^19^ Assuming an enthalpy-dominated free-energy barrier (i.e., Δ*S* ^‡^ ≈ 0 due to Δ*x* ^‡^ = 0.046 nm), we determined the height of the free energy barrier (Δ*G* ^‡^ ≈ 40 *k*_B_ *T*) and thereby the dissociation attempt frequency (*A* ≈ 6 × 10^10^ s^–1^). Combining our constant-velocity and constant-force data at 20 °C, we measured average lifetimes over six decades, during which SdrG:Fgβ behaved as a slip bond. We then repeated this assay at 20 °C for the adhesin-ligand pair SdrE:CFH. In contrast with the recent single-cell findings,^18^ we found no evidence of a high-force catch-bond–slip-bond transition in the 800–2200 pN range. Rather, we again observed slip-bond behavior over six decades of lifetimes.

## Results and Discussion

### A Mechanically Robust Single-Molecule Adhesin-Ligand Assay Using Site-Specific Anchoring

Our surface conjugation procedure follows the protocol developed in previous work,^7^ where the protein constructs had a terminal ybbR tag,^20^ a short 11-aa sequence that can be enzymatically conjugated to coenzyme-A (CoA). To ensure the proper pulling geometry, the ybbR-tag was placed on the C-terminal end of the adhesins, which was then conjugated to a CoA-derivatized, PEG-coated AFM cantilever [BioLever Mini, Olympus; *k*_meas_ = 129–141 pN/nm (Table S1)]. The ligand-containing polyproteins started with either Fgβ or CFH at its N-terminus followed by ddFLN4, a marker protein,^21^ and ended with a C-terminal ybbR. Each polyprotein was enzymatically conjugated to CoA-derivatized, PEG-coated glass coverslips. PEG-coating the cantilever and the coverslip minimized unwanted tip-surface adhesion and nonspecific protein deposition.^22^ The full details of the current surface-conjugation protocol were recently reported^23^ and is described in Methods, including two modifications to our previous protocol for improved reliability and reduction in contour length variability due to the polydispersity in PEG length.

The assay was initiated by gently pressing the adhesin-coated cantilever into a polyprotein-coated glass coverslip (∼50–100 pN for 10 ms) followed by retraction at a constant velocity. Force, as measured by cantilever deflection, was digitized at 50 kHz. Figure 1D shows a typical force-extension curve for the SdrG:Fgβ assay with the initial unfolding of ddFLN4 followed by a high rupture force event (*F* > 2000 pN). The characteristic double-peaked unfolding signature of ddFLN4 ^21^ was used to verify single-molecule attachment (Figure 1D, inset).

Given SdrG:Fgβ’s rupture forces are on par with covalent bonds, we first wanted to verify that these events arose from the rupture of SdrG:Fgβ, rather than another element in our attachment scheme (Figure S1). To do so, we collected over 10,000 force-extension curves at 20 °C using a single cantilever [with over 3,000 events representing single molecules (Figure 2, left)]. The functional longevity of the AFM cantilever demonstrated that we were not breaking the covalently anchored SdrG molecules off the limited surface-area at the tip of the cantilever, as that would rapidly lead to a stripped tip incapable of forming a new SdrG:Fgβ complex. To improve data-acquisition efficiency and demonstrate that we were not mechanically breaking the polyprotein’s covalent attachment to the coverslip, we collected multiple force-extension curves at the same surface location (rather than a typical raster scan where each x-y location on a grid is probed only once or a few times). By using this scheme, we collected over 800 single-molecule force-extension curves from the same spot. Given the low surface concentration (∼1% attachment frequency), this suggests an individual surface-anchored polyprotein could be probed hundreds of times, verifying the polyprotein’s surface conjugation was robust (see Methods for details). Together, these results provided confidence in interpreting the high-force rupture events as the rupture of the SdrG:Fgβ adhesin-ligand pair.

**Figure 2:**
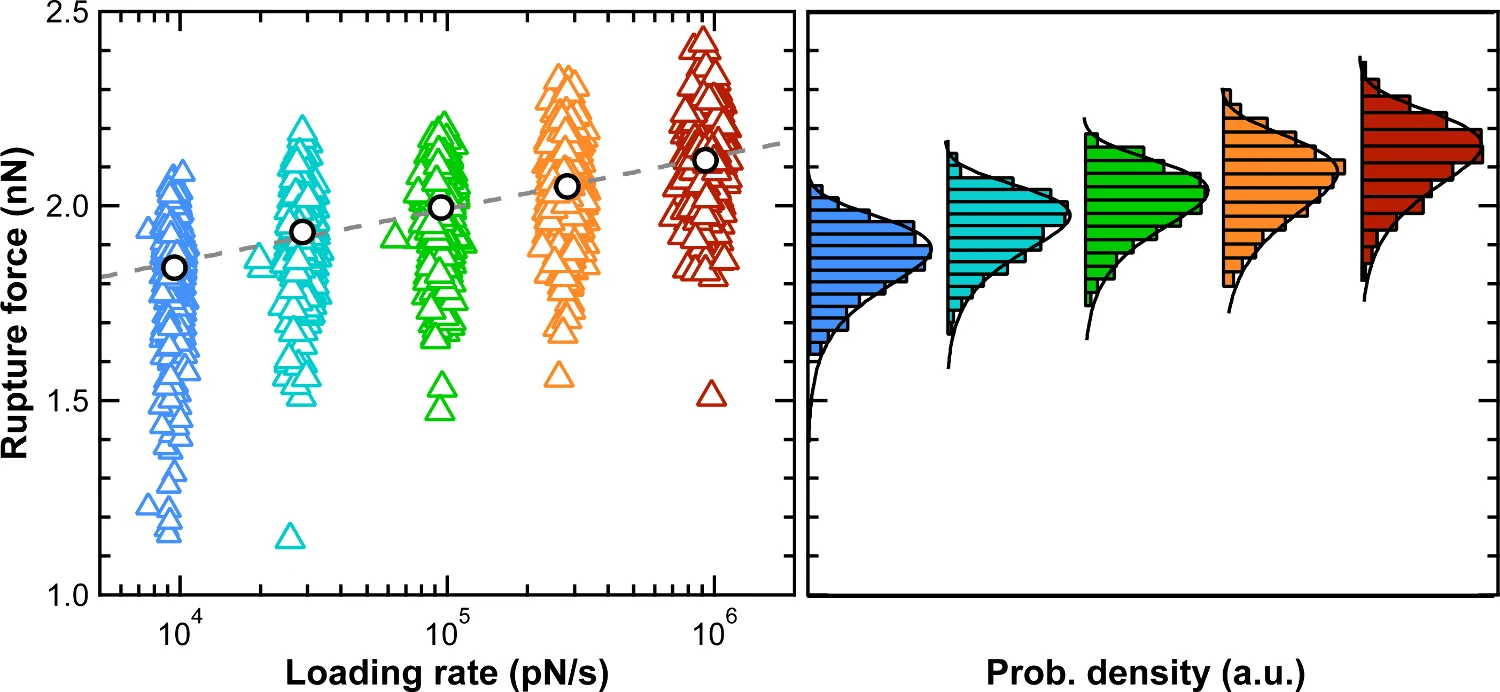
Rupture force of SdrG:Fgβ probed over a range of loading rates (∂F/∂t) in the constant-velocity assay. (Left) Triangles represent individual rupture force measurements, while circles represent the average for each loading rate. Different velocities represented by different colors: 100 nm/s (9,510 pN/s, blue), 300 nm/s (28,700 pN/s, teal), 1 μm/s (94,800 pN/s, green), 3 μm/s (282,000 pN/s, orange) and 10 μm/s (926,000 pN/s, red). A Bell–Evans fit to the average rupture plotted in gray and yielded Δx^‡^ = 0.070 ± 0.005 nm (fit ± SD) and log­(*k*_0_) = −11.5 ± 0.8. (Right) Rupture force distributions (*N* = 932, 715, 666, 585, 439, left to right respectively) individually fit to the Dudko–Hummer–Szabo model (black line) illustrates the expected asymmetric distribution. For clarity, bins with *N* *<* 3 are not shown. All of the data was acquired with a single cantilever demonstrating the robustness of the surface-anchoring scheme and providing for improved precision^24^ by avoiding uncertainties associated with comparing distributions acquired with different cantilevers.

### Constant-Velocity Assay Yields Δ*x* ^‡^ and Asymmetric Rupture Force Distributions

We varied the retraction velocity (*v* = 0.1–10 μm/s) to acquire a dynamic force spectrum with >400 individual events at each velocity (see Supporting Information for details of the data-acquisition software). We plotted the resulting individual events and the mean rupture force at each retraction velocity as a function of the loading rate (Figure 2, left). A Bell–Evans model^10,11^ fit to the average rupture force yielded Δ*x* ^‡^ = 0.070 ± 0.005 nm (fit ± SD of fit) and *k*_0_ = 3 ± 6 × 10^–12^ s^–1^, similar to prior results (Δ*x* ^‡^ = 0.051 nm, *k*_0_ = 9.2 × 10^–11^ s^–1^).^7^ [Note, errors for fits are reported as ± SD, where the standard deviation is the square root of the diagonals of the covariance matrix.] This agreement supports our conclusion that our assay is probing the nanomechanics of SdrG:Fgβ. This very small Δ*x* ^‡^ is on par with the Bohr radius of a hydrogen atom (0.053 nm). It represents an extremely “brittle” interaction arising from the requirement to simultaneously break multiple hydrogen bonds. We also note that being able to quantitatively describe this dynamic force spectrum, with its log-linear increase in rupture force with increasing loading rate, with the Bell–Evans model^10,11^ indicates a slip-bond mechanism, as it is the physical mechanism underlying the model.

The resulting rupture force probability distributions were asymmetric, as predicted by both the Bell–Evans and the DHS models (Figure 2, right).^10,19^ Observing this asymmetry is an indication of the overall data quality and was aided by taking all the data with a single cantilever for improved precision, avoiding uncertainties associated with comparing distributions acquired with different cantilevers.^24^ While asymmetric rupture force probability distributions are observed occasionally in optical-trapping data, it is still relatively rare in AFM studies, with some notable exceptions in both early^25^ and more contemporary studies.^7^ We highlighted this expected asymmetry by fitting the individual measured rupture force distributions to the DHS model, which relaxes the assumptions of the Bell–Evans by using an additional parameter, an explicit energy barrier landscape shape, that leads to the effective distance to the transition state decreasing with increasing force. However, we note that the data presented in Figure 2 does not sufficiently constrain the DHS model to yield a measured Δ*G* ^‡^, which typically requires observing curvature in the dynamic force spectrum (See Methods). For SdrG:Fgβ, such curvature has only been observed *in silico* via all-atom steered MD simulations at extremely high pulling speeds (*v* ≥ 2 m/s).^7^

### A High-Throughput, Constant-Force AFM-Based Assay

Having established confidence in our assay and concurrence with prior results, we switched to the more physiologically relevant constant-force assay. Constant-force single-molecule assays were originally developed for optical-trapping-based studies of molecular motors^26^ and RNA folding.^27^ In AFM studies,^28^ constant-force assays can directly measure lifetime (τ) at a given force (Figure 1F). We initialized the constant-force assay using the same methodology as the above constant-velocity assay. Upon reaching 200 pN, a feedback-loop was activated that rapidly brought the SdrG:Fgβ complex to the target force in under 10 ms with a sampling rate of 1 kHz. As shown in Figure 1F, the lifetime was measured from once the target force was reached until a rupture event was observed. As in the constant-velocity assay, the unfolding signature of ddFLN4 (before the feedback loop activated) was used to verify single-molecule attachment.

In a constant-force assay using feedback, it is critical to understand force drift. Drift in the zero-force position of the cantilever^29^ leads to an unmeasured change in the applied force.^30^ To quantify this drift, we periodically measured cantilever deflection for 5 min at 300 nm over the surface prior to each experiment, proceeding only when the drift rate was ≤16 pN/min, which typically required >3 h (Figure S2). We used the long-time portion of the Allan deviation—a plot of force precision as a function of averaging time—to determine the linear drift rate. We then set the minimum applied constant force under each condition such that essentially all individual lifetime measurements (>99.7%) did not experience a force drift exceeding 5%, with the vast majority of them experiencing significantly less drift. It is the rare long-lived event within an exponential distribution that sets this limit. This methodology, coupled with the high sustained forces (>1000 pN) is what enabled measuring lifetimes under essentially constant force over extended periods (>60 s) with confidence on a commercial AFM with a commercial AFM cantilever.

By pulling on the same polyprotein multiple times, we enhanced the data-acquisition rate, allowing us to acquire a large number of individual lifetime measurements efficiently. Figure 3A shows 25% of the records taken under 1400 pN of force at 20 °C. We next computed the cumulative probability distributions, where each data point in a cumulative probability distribution-versus-time plot represents the probability of the SdrG:Fgβ interaction surviving up to the given time. Each individual lifetime measurement is represented in these probability distributions, which avoids binning. This distribution was well fit to a single exponential and yielded τ_fit_ (Figure 3B, *N* = 165). This result indicates that ligand dissociation is governed by a single rate-limiting kinetic step. The uncertainty in τ_fit_—and therefore the off rate (*k*_off_ = 1/τ_fit_)—was determined as the standard deviation from a bootstrap analysis.^31^ The resulting uncertainties averaged 13% (Table S2), in rough agreement with simple counting statistics $(∼1/\sqrt{N}$ where *N* is the number of samples). We then repeated this process over a range of forces (1200–1800 pN, Figure 3C). To highlight the exceptional mechanical stability of the SdrG:Fgβ interaction, our average lifetime under 1200 pN of force was 20 ± 2 s. For comparison, streptavidin–biotin is computed to have a similar lifetime at a load of just ∼260 pN.^32^

**Figure 3:**
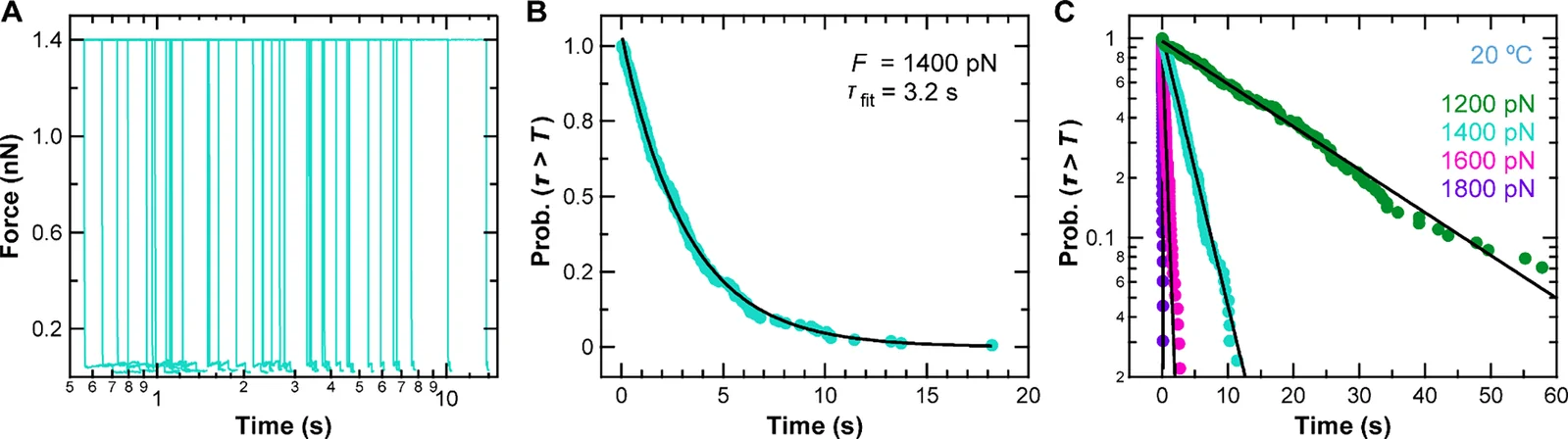
Analysis method for the constant-force assay. (**A**) Representative traces for 25% of the lifetime measurements under a load of 1400 pN at 20 °C. Note, the short-lived low-force (∼30 pN) plateau after the high-force ruptures arises from hydrodynamic drag due to the cantilever being rapidly retracted to its limit after ligand dissociation as the feedback loop tries to maintain a constant force. (**B**) Normalized cumulative probability distribution of the measured lifetimes fit to a single exponential. (**C**) Logarithmic plot of the normalized cumulative probability distributions versus time measured at 20 °C for 1800 pN [purple, *N* = 66, τ_fit_ = 41 ± 4.3 ms (fit ± SD)], 1600 pN (pink, *N* = 136, τ_fit_ = 0.508 ± 0.054 s), 1400 pN (turquoise, *N* = 165, τ_fit_ = 3.21 ± 0.23 s), and 1200 pN (green, *N* = 127, τ_fit_ = 20.2 ± 2.0 s), from left to right, respectively, with accompanying fits to a single exponential. All of the data were acquired with a single cantilever.

### SdrG:Fgβ Lifetimes as a Function of Force (1000–1800 pN) and Temperature (10–37 °C)

To measure the unbinding kinetics at the physiologically relevant temperature of 37 °C and, more generally, explore the temperature dependence of SdrG:Fgβ under constant force, we repeated this data-acquisition scheme over a range of temperatures (10, 20, 30, and 37 °C). Minimum forces were chosen to try to keep individual measurements of τ below ∼180 s (limited by the force drift). The maximum force applied was chosen to keep the measured average τ above ∼50 ms (limited by time to reach the set point and the digitization rate). Over all conditions, the resulting cumulative probability distributions continued to be well described by a single exponential, as demonstrated by the cumulative probability distributions for four different forces at 37 °C (Figure 4A). Table S2 catalogs τ_fit_ over all the temperatures and forces.

**Figure 4:**
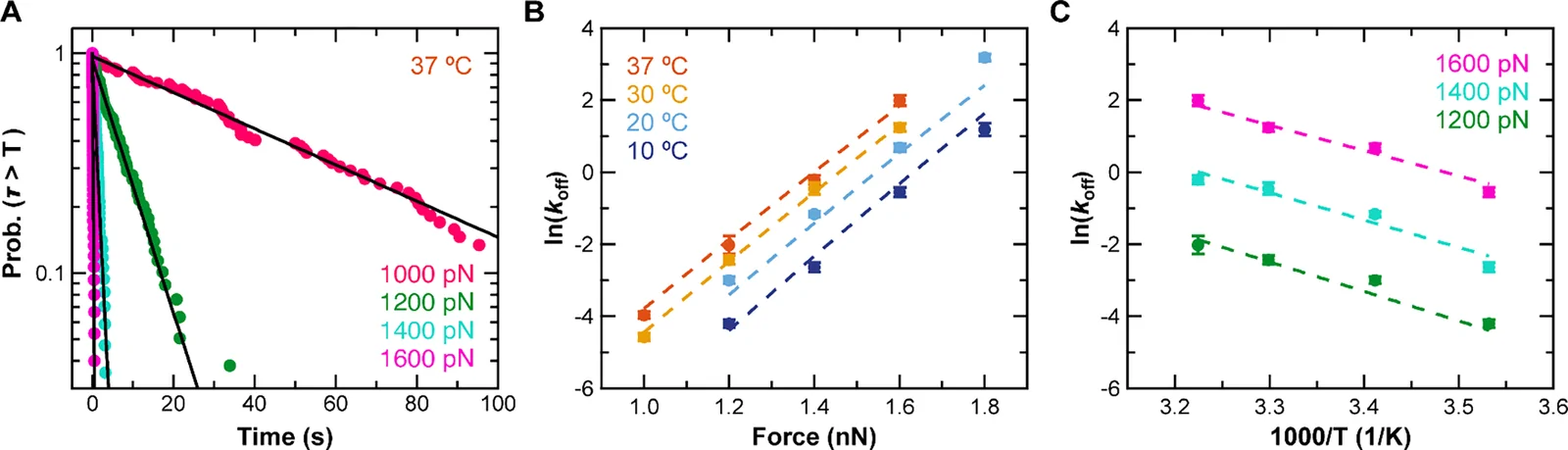
Kinetic and thermodynamic analysis of constant-force data. (**A**) Logarithmic plot of the normalized cumulative probability distributions measured at 37 °C for 1600 pN (pink, *N* = 75, τ_fit_ = 0.137 ± 0.019 s), 1400 pN (turquoise, *N* = 78, τ_fit_ = 1.22 ± 0.15 s), 1200 pN (green, *N* = 79, τ_fit_ = 7.5 ± 1.9 s), and 1000 pN (magenta, *N* = 80 τ_fit_ = 53.0 ± 5.0 s), from left to right, respectively. (**B**) Natural logarithm of the off rate (*k*_off_ = 1/τ_fit_) plotted as a function of force across a range of temperatures: 37 °C (orange), 30 °C (gold), 20 °C (light blue), 10 °C (dark blue). (**C**) Natural logarithm of *k*_off_ plotted versus 1/*T* over a range of forces 1600 pN (pink), 1400 pN (turquoise), and 1200 pN (green) from top to bottom, respectively, where *T* is absolute temperature. Error bars in panels B and C were calculated by bootstrap analysis, but in some cases are smaller than the symbol size. Dashed lines in both (B) and (C) represent a single global fit to all data using the Dudko–Hammer–Szabo (DHS) model, giving a distance to the transition state of Δ*x* ^‡^ = 0.046 ± 0.013 nm and an enthalpic barrier height of Δ*H* ^‡^ = 40 ± 4 *k*_B_ *T*.

In agreement with the Bell model,^11^ *k*_off_ increased exponentially with increasing *F* over all explored temperatures [*k*_off_(*F*) ∝ exp­(*F*Δ*x* ^‡^/*k*_B_ *T*)] (Figure 4B), where *k*_B_ *T* is the thermal energy. As expected, off rates increased with increasing temperature. The similarity of the slope for each particular temperature data set—when plotted as ln­(*k*_off_)-versus-*F*—indicates no substantial change in Δ*x* ^‡^, since the slope is given by Δ*x* ^‡^/*k*_B_ *T* within the Bell model. Individual fits did not show a consistent linear increase in Δ*x* ^‡^ with temperature (Figure S3) and only a mere 13% change overall. We note that essentially no change in Δ*x* ^‡^ over this temperature range is a nontrivial result. Prior AFM characterization for the unfolding of a protein (ddFLN4) showed a temperature-dependent softening in Δ*x* ^‡^, with Δ*x* ^‡^ more than doubling between 10 and 37 °C (0.34 ± 0.05 nm and 0.78 ± 0.05 nm, respectively).^25^

### Thermodynamic Analysis Yields the Enthalpic Dissociation Barrier Height (Δ*H* ^‡^ = 40 *k*_B_ *T*)

An alternative way to view the data is with an Arrhenius plot ln­(*k*_off_) versus 1/*T* (Figure 4C). To conceptualize this framework, we start with $k_{0}=Ae^{-\Delta G^{‡}/k_{B}T}$ , where *A* is the attempt frequency and then add in the Bell model’s force dependence to yield $k_{\mathrm{off}}=Ae^{-\Delta G^{‡}+F\Delta x^{‡}/k_{B}T}$ . We substitute with Δ*G* ^‡^ = Δ*H* ^‡^ – *T*Δ*S* ^‡^, where Δ*S* ^‡^ is the change in entropy between the bound and transition states. We next rearrange the formula to match the functional dependence ln­(*k*_off_) vs 1/*T* shown in Figure 4C, which yields

1 $$\ln\left(k_{\mathrm{off}}\right)=\left[\ln\left(A\right)+\frac{\Delta S^{‡}}{k_{B}}\right]-\frac{1}{T}\left[\frac{1}{k_{B}}\left(\Delta H^{‡}-F\Delta x^{‡}\right)\right]$$

Equation 1 therefore shows the slope of an Arrhenius plot is given by (Δ*H* ^‡^ – *F*Δ*x* ^‡^)/*k*_B_ while the y-intercept is given by a combination of *A* and Δ*S*. Therefore, it is possible to determine Δ*H* ^‡^ from our data given that *F*, Δ*x* ^‡^, and *k*_B_ are known. However, for a given *y*-intercept, Δ*S* ^‡^ and *A* are not individually determined but their relationship is constrained.

Having established that a similar set of parameters were describing individual aspects of the overall constant-force data set, we sought to do a global fit to the data shown in Figure 4B and C. To do so, we used the more realistic DHS model instead of the Bell model. As a reminder, the DHS model relaxes the Bell model’s assumption of a force-independent barrier position by using an explicit energy-landscape shape, in this case a linear cubic potential, that leads to the transition state position decreasing with increasing force. We note that the DHS model assumes a high-barrier approximation, and therefore starts to fail near the critical force (*F*_c_ = 3Δ*G* ^‡^/2Δ*x* ^‡^).^19^ We will go onto show that all our constant-force data is well below this critical force.

To better isolate the thermodynamics of force-induced ligand dissociation for SdrG:Fgβ, we separated the enthalpic and entropic components of the Gibbs free energy in the DHS model, as done above for the Arrhenius analysis. For the DHS model, this gives

2 $$k_{\mathrm{off}}\left(F\right)=k_{0}{\left(1-\frac{2F\Delta x^{‡}}{3\Delta G^{‡}}\right)}^{1/2}e^{\Delta G^{‡}/k_{B}T\left[1-{\left(1-2F\Delta x^{‡}/3\Delta G^{‡}\right)}^{3/2}\right]}$$

3 $$k_{0}=Ae^{-\Delta G^{‡}/k_{B}T},\;\mathrm{and}$$

4 $$\Delta G^{‡}=\Delta H^{‡}-T\Delta S^{‡}$$

We then did a global fit to the data in Figure 4B and C using a least-squares fitting algorithm where there were four free parameters: Δ*x* ^‡^, Δ*H* ^‡^, *A*, and Δ*S* ^‡^ (Figure 4 B,C; dashed lines).

Using this methodology, we determined well-constrained values for Δ*x* ^‡^ (0.046 ± 0.013 nm) and Δ*H* ^‡^ (40 ± 4 *k*_B_ *T*) when using a linear-cubic shaped potential, where the values are fit ± SD. We note that when fitting with a cusp-shaped potential instead, the fitted values were essentially unchanged [Δ*x* ^‡^ = 0.050 ± 0.014 nm and Δ*H* ^‡^ = 41 ± 4 *k*_B_ *T*]. To explore how the fitting results depended on starting parameters, we explored a feasible parameter space (see Methods for details). The fitted values for Δ*x* ^‡^ and Δ*H* ^‡^ were independent of the starting and final values for Δ*S* ^‡^ and *A*, which were not well constrained. The standard deviation of the fit for both Δ*S* ^‡^ and *A* far exceeded their values, indicating the curvature in minimizing χ^2^ with respect to those fitting parameters was shallow. To check this, we calculated χ^2^ values over a range of Δ*S* ^‡^ and *A*, where Δ*x* ^‡^ and Δ*H* ^‡^ were fixed at their fitted values. The resulting χ^2^-space had a clear minimum-trench (Figure S4), indicating that while the values for Δ*S* ^‡^ and *A* where not individually constrained, the data does constrain the numerical relationship between Δ*S* ^‡^ and *A* such that Δ*S* ^‡^ ≈ 8.7 × 10^–2^ – 8.1 × 10^–3^×log­(*A*). Thus, a particular choice of Δ*S* ^‡^ leads to a particular value of *A*.

### Estimating the Dissociation Attempt Frequency

We next considered a structural argument to make a reasonable approximation for Δ*S* ^‡^ and thereby *A*. Steered MD simulations show that the binding pocket of the SdrG:Fgβ complex is extremely structured, such that lateral movement of Fgβ is highly restricted, and the backbone “never bends nor buckles”.^7^ Additionally, the distance between the bound state and the transition state—exactly what is measured by Δ*x* ^‡^—is a mere ∼0.05 nm along the direction of force, a displacement approximately equal to the radius of a hydrogen atom. We therefore assert that the conformational phase space of the complex is essentially unchanged between the bound and the transition states and thus we estimated Δ*S* ^‡^ = 0. Under this estimate, we calculated Δ*G* ^‡^ = 40 ± 2 *k*_B_ *T* and log­(*A*) = 10.8 ± 0.7.

Our estimate for the attempt frequency is 6 × 10^10^ s^–1^ under the assumptions outlined above. An estimate of attempt frequency for an individual hydrogen bond in an α-helix is ∼5 × 10^11^ s^–1^ as derived from simulations.^33^ It seems reasonable that this value would act as an upper bound for the attempt frequency of a ligand bound primarily through multiple hydrogen bonds. While this value for *A* may be extreme in comparison to estimates for protein folding^34^ of 10^6^–10^7^ s^–1^, we note that the unique structure of the SdrG:Fgβ complex, the very high protein-ligand tension applied in the present study, and the 1D motion of the ligand in this linear binding pocket all likely impact *A*.

A Δ*G* ^‡^ of 40 *k*_B_ *T* highlights the extraordinary mechanical stability of SdrG:Fgβ. However, this value is lower than the 66 *k*_B_ *T* reported by Milles et al.^7^ in a fit to a data set that combined AFM measurements with all-atom, steered MD simulations pulling up to 12.5 m/s.^7^ A portion of this discrepancy may arise from the calibration of cantilever stiffness, which is prone to ∼15% error^35^ or even higher^36^ in interlaboratory, round-robin comparisons using the standard thermal method. Consistent with this, our rupture forces are ∼20% lower than that reported by Milles et al., which could partially explain the lower measured energy barrier. Alternatively, one could attribute this difference to our approximation of Δ*S* ^‡^, as a larger, negative entropic barrier would lead to a higher Δ*G* ^‡^. We note that, if we shift away from our estimate of Δ*S* ^‡^ = 0 to even a modest value for *T*Δ*S* ^‡^ (≈−2 *k*_B_ *T*) leading to Δ*G* ^‡^ = 42 *k*_B_ *T*, the attempt frequency would exceed 5 × 10^11^, given the above relationship for Δ*S* ^‡^ and *A* (Figure S4). We regard such a value as unphysical, given the aforementioned attempt frequency for an individual hydrogen bond in an α-helix (∼5 × 10^11^ s^–1^).^33^ It is also possible that some portion of this difference in Δ*G* ^‡^ is physically real, and the energetic parameters change slightly in the much higher force range (3000–5000 pN) that were explored by Milles et al.’s MD simulations.^7^ We also note that the higher values of these simulated forces are reaching our estimate of 5200 pN for the critical force (*F*_c_ = 3Δ*G* ^‡^/2Δ*x* ^‡^).

### SdrG:Fgβ and SdrE:CFH Are Both High-Force Slip Bonds over Six Decades of Lifetimes

We first show that the SdrG:Fgβ interaction acted as a slip bond over the entire force range probed (1200–2400 pN) and spanned six-decades of lifetimes (Figure 5A). To do so, we combined our constant-velocity and constant-force data at 20 °C onto a single log-linear plot of τ-versus-*F* using a transformation developed by Dudko, Hummer, and Szabo.^19^ This transformation converted rupture force distributions into force-dependent lifetimes (See Methods for details). As shown in Figure 5A, the data collapsed nicely onto a single master curve, with significant overlap between the data. The curve shows no deviation from the expected log-linear dependence of lifetime vs force. This result signifies that while a catch-bond force-dissociation pathway exists at low forces (<30 pN),^15^ the slip-bond dissociation pathway^7,16^ dominates in the force range explored here.

**Figure 5:**
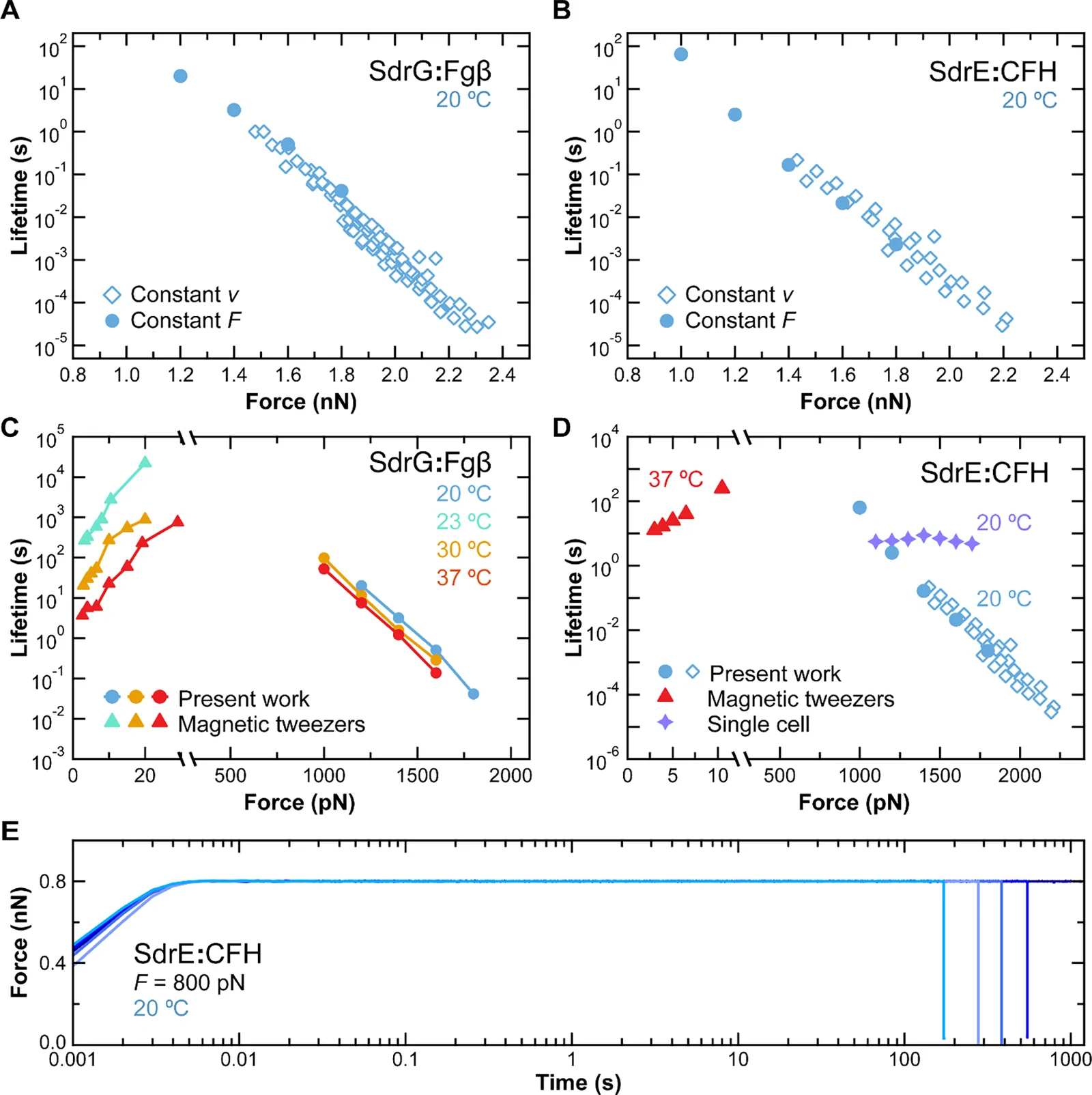
Lifetime measurements for two adhesin-ligand pairs. (**A**) SdrG:Fgβ lifetimes plotted as a function of force deduced by combining constant-force data (dark circles) and constant-velocity data (open diamonds), showing slip bond behavior over six decades of lifetime. (**B**) The same plot for the SdrE:CFH interaction. The constant-velocity data were transformed to lifetime using the measured rupture force distributions and an algorithm introduced by Dudko, Hummer, and Szabo.^48^ (**C**) A comparison of SdrG:Fgβ lifetime measurements by AFM (present work, circles) and magnetic tweezers^15^ (triangles) for 20 °C (light blue), 23 °C (turquoise), 30 °C (gold), and 37 °C (red). (**D**) A comparison SdrE:CFH lifetime measurements for single-molecule AFM (present work, light blue circles/open diamonds), single-cell AFM^18^ (purple stars), and single-molecule magnetic tweezers^15^ (red triangles). Note, the horizontal force-axis breaks are used when comparing magnetic tweezers and AFM data to clearly display each data set. (**E**) Example SdrE:CFH lifetime measurements under 800 pN of load at 20 °C are shown, with lifetimes of 172 s, 278 s, 381 s, 546 s, and >1200 s. Note, five out 11 800-pN traces exceeded 1200 s.

Finally, we confirmed that the observed high-force slip-bond behavior is not unique to SdrG:Fgβ by utilizing the same assay to probe a second adhesin-ligand pair (SdrE:CFH) at 20 °C, the same adhesin-ligand pair previously reported to have a catch-bond–slip-bond transition at 1400 pN @ 20 °C in the single-cell assay.^18^ A dynamic force-spectrum analysis taken at constant velocity again showed a log-linear increase in the rupture force with increasing loading rate (Figure S5), while the constant-force data yielded exponential lifetime distributions (Figure S6A,B, Table S2). For the latter data set, we again observed an exponential increase in *k*_off_ with increasing force, consistent with slip-bond mechanics (Figure S6C). A Bell model analysis yielded Δ*x* ^‡^ = 0.049 ± 0.003 nm (fit ± SD). Combining the constant-force and the constant-velocity data sets shows that the SdrE:CFH interaction exhibited slip-bond behavior over the entire force range measured (1–2.2 nN) covering over six decades of lifetimes (Figure 5B).

### Comparison with Prior Studies of SdrG:Fgβ and SdrE:CFH under Constant Force

We next sought to put our work into perspective of previous studies of SdrG:Fgβ and SdrE:CFH (Figure 5C,D, respectively). When combined with low-force measurements made by magnetic tweezers,^15^ our data fits well into the proposed two-state model for adhesin-ligand dissociation.^15,16^ For SdrG:Fgβ, data taken across various temperatures suggests the existence of a catch-bond–slip-bond transition somewhere in the 60–1000 pN range (Figure 5C), with the authors of the magnetic tweezer study estimating a transition at >300 pN.^15^ While the data presented in this work for SdrE:CFH is at a different temperature than reported in the magnetic-tweezer study^15^ (20 °C vs 37 °C, respectively), the combined data again shows an exponentially increasing lifetime with force in the low force regime (3–10.5 pN) and an exponentially decreasing lifetime in the high force regime (1–2.2 nN) (Figure 5D).

Given this catch-bond behavior in low-force regime and slip-bond behavior in the high-force regime (Figure 5C,D), we expect that these bacterial adhesins are best described as a one-state, two-pathway catch-bond–slip-bond system.^37^ First, each pathway is well-described by a distinct Δ*x* ^‡^ deduced by analyzing several decades of force-dependent lifetimes [e.g., Δ*x* ^‡^ = −1.20 ± 0.07 nm for the low-force catch-bond pathway for SdrG:Fgβ^15^ and Δ*x* ^‡^ = 0.046 ± 0.013 nm for its high-force slip-bond pathway (present study and prior constant-velocity data^7^)]. Second, there is a well-defined bound state in both structural^17^ and computational^7^ studies. Third, both experiments and computational studies detail two distinct dissociation pathways.^7,15,16^ Given this, we expect there to be a smooth transition between these pathways as the lifetimes of each force-dependent pathway become comparable.

Our single-molecule, *in vitro* SdrE:CFH data stands in contrast to the recent single-cell force spectroscopy data^18^ that reported a catch-bond–slip-bond transition for a single SdrE:FH complex at 1400 pN (Figure 5D, purple stars). On the logarithmic scale of the *y*-axis in Figure 5C, the single-cell data is relatively flat as a function of force with a small peak at 1400 pN. In contrast, our data for SdrE:CFH shows a uniform line on the log-linear plot described by a single physical mechanism—a slip bond—over six decades of lifetimes for a force range that spans 1400 pN (Figure 5D, blue circles and diamonds), in mechanistic agreement with our SdrG:Fgβ results. We note that the data presented here utilizes a fully covalent, site-specific conjugation scheme—leading to a well-defined pulling geometry—and a marker protein (ddFLN4) to ensure only single-molecule attachments were analyzed.

While we do not know the exact origin of this discrepancy, the single-cell assay presents several challenges to precise mechanistic interpretation. A recombinant segment of factor H (FH, aa 860–1231) was covalently anchored to the AFM tip though through a nonsite-specific, surface-exposed amine–based conjugation scheme.^18^ Variability in FH-tip attachment may be the origin of the observed variable mechanical fingerprints and why the data showed both low-force and high-force rupture events, a result not seen in our data. To test this idea, we applied force to the N-terminus of the CFH ligand (Figure S7A), rather than its C-terminus as used when pulling in the canonical high-force loading geometry^7^ (Figure 1B). As previously reported for SdrG:Fgβ,^7^ this noncanonical pulling geometry resulted in a dramatically lower rupture force [208 ± 30 pN (mean ± S.D.; *N* = 57) @ *v* = 1 μm/s], with no observed high-force rupture events (Figure S7A). In contrast, the same individual SdrE-coated cantilever used to acquire the noncanonical pulling geometry yielded a rupture force of 2000 ± 220 pN (mean ± S.D.; *N* = 330) @ *v* = 1 μm/s] in the canonical pulling geometry with only 0.6% (*N* = 2) of curves exhibiting a rupture force <1500 pN (Figure S7B). These results suggest that observed low-force and high-force rupture events in the single-cell data may arise, in part, from pulling in the noncanonical and canonical geometries due to nonsite-specific protein conjugation.

Additionally, without a marker protein, the single-cell data does not have a definitive signature of studying a single molecule. With a reported average tip-attachment frequency of 47%, application of simple Poisson statistics suggests 28% of attachments arose from pulling multiple molecules in parallel, an analysis introduced in one of the earliest single-molecule force-spectroscopy papers.^38^ We note that future single-cell assays—needed for studying adhesin-ligand complexes in a broader biological setting—could bypass these concerns by using a site-specific anchoring scheme where the protein of choice, a recombinant FH in the present discussion, is embedded into a larger polyprotein construct with a marker protein. This polyprotein would then be site-specifically anchored to the AFM tip, a strategy used in previous *in vitro* AFM studies.^39,40^ Finally, in the single-cell studies, there are several types of covalent bonds along the force-propagation pathway with potentially different rupture forces. In the present *in vitro* assay, the collection of 1000s of individual rupture forces from a single tip and 100s from a single individual surface-anchored polyprotein unambiguously demonstrates the mechanical robustness of our surface-conjugation scheme and allows us to assign the measured rupture to the force-induced dissociation of the adhesin-ligand complex.

### Individual Lifetimes of SdrE:CFH at 800 pN Exceed 15 min

Finally, after overnight incubation, the instrumental drift rate for the SdrE:CFH experiment dropped from 11 pN/min to 4 pN/min (Figure S8), facilitating limited data collection at 800 pN (examples in Figure 5E). Four out of 11 measurements at 800 pN had lifetimes exceeding 15 min. This data, albeit incomplete, indicates that slip-bond behavior continues down to at least 800 pN. As a scientific note, this data with a polyprotein containing ∼160 unstructured, amide bonds in the force-propagation pathway after the unfolding of ddFLN4 strongly suggests that prior work^41^ reporting an amide bond lifetime of 0.171 ± 0.025 s under 800 pN of load was observing the rupture of a different bond in their assay.

### Study Limitations

The extraordinary lifetime of these adhesin-ligand pairs (Figure 5C,D) prevented direct detection of the catch-bond—slip-bond transition. Based on the magnetic-tweezer data, the expected transition will occur at times significantly longer than >6 h.^15^ In contrast, a catch-bond–slip-bond transition has been observed in other biological systems where peak-lifetimes are more experimentally accessible, including ∼0.03 s for actomyosin-ADP,^42^ ∼0.08 s for L-selectin–P-selectin glycoprotein ligand-1,^29,43,44^ ∼1.2 s for P-selectin–P-selectin glycoprotein ligand-1,^45^ and ∼12 s for vinculin–F-actin.^46^ For the AFM-based assay used here, this limitation arises from the force stability of our cantilever (Olympus BioLever Mini) (Figure S2,S8). There is, therefore, an ongoing need to measure the catch-bond–slip-bond transition for adhesin-ligand pairs in the unexplored range of ∼50–800 pN. Probing this force range will require force-spectroscopy techniques with exceptionally low force drift over lifetimes exceeding 6 h,^15^ with one potential solution being modified AFM cantilevers that exhibit minimal force drift.^29,43,44^

Exploring a temperature range beyond 10–37 °C would provide increased confidence in the reported thermodynamic parameters. However, we experienced condensation at temperatures below 10 °C, an issue that could potentially be addressed by using dry N_2_. Above 37 °C, the evaporation rate from the AFM’s fluidic cell prevented acquiring large data sets over extended periods. In future studies, the use of a perfusion cantilever holder—which allows for *in situ* buffer exchange—may address this issue.

While we believe our structural argument for Δ*S* ^‡^ ≈ 0 is reasonable given Δ*x* ^‡^ = 0.046 ± 0.013 nm, we were unable to independently determine Δ*S* ^‡^ and *A* without this assumption. This limitation arises from the lack of observed curvature in the ln­(*k*_off_)-vs-*F* plot (Figure 4B), preventing deduction of Δ*G* ^‡^. Molecular dynamics simulations have observed the related curvature in the dynamic force spectrum of SdrG:Fgβ near their highest pulling speeds (*v* ≥ 2 m/s);^7^ however, this regime is not currently accessible by our AFM, though we note that such high pulling speed have been achieved using high-speed AFMs.^47^

## Conclusions

We explored the high-force regime of the bacterial adhesin-ligand complex SdrG:Fgβ using both constant-force and constant-velocity assays, collecting over 4,400 single-molecule records. The constant-force data—designed to better mimic physiological force application—was taken over a range forces (1000–1800 pN) and temperatures (10–37 °C). The SdrG:Fgβ complex continued to show exceptional stability, highlighted by an average lifetime over 50 s under 1000 pN of applied force at 37 °C. We quantified *k*_off_ as a function of both force and temperature and globally fit this data to a DHS model to extract free-energy landscape and thermodynamic parameters. Looking forward, we expect that the temperature-dependent force-clamp methodology used here could yield Δ*x* ^‡^, Δ*G* ^‡^, Δ*H* ^‡^, Δ*S* ^‡^, and *A* within the DHS model^19,48^ for systems^49^ exhibiting the expected curvature in a plot of ln­(*k*_off_)-vs-*F*. Lastly, we confirmed that SdrE:CFH also exhibits slip-bond behavior in the high-force regime (800–2200 pN), indicating this behavior is not unique to SdrG:Fgβ.

## Methods

### Single-Molecule Construct Preparation

The adhesin constructs [SdrG­(N2,N3)-His-ybbR and SdrE­(N2,N3)-His-ybbR-sortase] and ligand-terminated polyprotein constructs (Fgβ-ddFLN4-His-ybbR, CFH-ddFLN4-His-ybbR, and ybbR-his-α_3_D-CFH) were expressed in competent *E. coli* (BL21-DE3). Note, for investigating SdrE:CFH in the noncanonical pulling geometry (Figure S7A), we used α_3_D as the marker protein due to its lower unfolding force^50^ to avoid overlap in the rupture force distributions of the marker protein ddFLN4 and SdrE:CFH in the noncanonical pulling geometry. Protein purification was done through standard methods using two Thermo Scientific PI88224 HisPur Ni-NTA mini spin columns (0.2 mL resin). We pooled and dialyzed the highest concentration samples, as measured by OD_280_ (Nanodrop), into a storage buffer [25 mM Tris-HCl (pH 7.4), 150 mM NaCl, and 1 mM TCEP]. The proteins were aliquoted and frozen at –80 °C until needed.

### Surface Functionalization and Protein Conjugation

We covalently and site-specifically anchored the surface protein constructs to commercial amine glass coverslips (PolyAn) using a protocol adapted from Milles et al.,^7,51^ where Sfp^52^ enzymatically anchored the ybbR tag to a CoA-derivatized, PEG-coated surface (Figure S1). For surface anchoring to coverslips, we coated commercial amine coverslips in Borate Buffer [50 mM boric acid, 12.5 mM NaOH (pH 8.5)] for 1 h, followed by incubation in TFP ester-PEG_12_-maleimide Solution [20 mM TFP-ester-PEG_12_-maleimide (Vector Laboratories) dissolved in 25% DMSO, 75% pH 8.0 Borate Buffer] for another 1 h. After PEGylation, we rinsed the slides in 1 L of water for at least 2 min and immediately incubated in CoA Solution [1 mM Coenzyme A, 2 mM TCEP, 50 mM Na_2_HPO_4_ (pH 7.2), 50 mM NaCl, 10 mM EDTA] overnight at 4 °C. After a final rinse in water, we dried and stored the coverslips under nitrogen at 4 °C for up to a month. Prior to experiments, we deposited a solution containing 0.3–2 μM polyprotein, 4 μM Sfp, and 20 mM MgCl_2_ in Assay Buffer [25 mM Tris-HCl (pH 7.5) and 150 mM NaCl] for 1 h, resulting in covalent linkage between the ybbR-tag and the CoA.^20^ Prior to loading into the AFM, we rinsed the surfaces in 20 mL of Assay Buffer by gently flowing buffer from a 0.2 μm-filtered syringe over the sample, removing excess buffer with a pipet tip connected to a vacuum line. Note, we replaced the oft-used NHS ester with TFP ester^23^ due to its higher stability, resulting in a lower hydrolysis rate.^53^

We covalently linked the adhesin proteins to AFM cantilevers using a similar protocol. Prior to functionalization, AFM cantilevers were cleaned by submersion for 2 min in subsequent 1 L beakers of water, isopropanol, and toluene, followed by another rinse in isopropanol. Cantilevers were then UV-ozone irradiated for 15 min prior to incubation in a solution containing 1 mL 100% ethanol, 1 mL 3-aminopropaldimethylethoxysilane (APDMES), and 5 μL water for 15 min. We then rinsed the cantilevers gently for 30 s in separate beakers of 90% ethanol (600 mL) and water (1 L) before placing them in a vacuum oven for 30 min at 80 °C. After allowing the cantilevers to cool to room temperature (∼5 min), we submerged them in Borate Buffer for 1 h, followed by deposition of 30–50 μL of TFP ester-PEG_12_-maleimide Solution for another 1 h. Finally, we rinsed the cantilevers in 1 L of water before submersing them in 30–50 μL droplets of CoA Solution for 1 h at room temperature. Cantilevers prepared this way could be stored for up to 1 week at 4 °C in CoA Solution or water. Prior to the experiment, cantilevers were rinsed for 2 min in a beaker containing 1 L of water and then submerged in a 20–30 μL droplet containing 40 μM adhesin, 4 μM SFP enzyme, and 20 mM MgCl_2_ in Assay Buffer for 1 h. We then rinsed cantilevers using 20 mL of Assay Buffer. All water was ultrapure.

We established confidence in the mechanical robustness of our surface-conjugation scheme by repeated acquisition of multiple force-extension curves taken at the same x-y location on the coverslip. For example, at one specific location, we collected over >1100 curves. Of these curves, 79% were single-molecule measurements (*N* = 894), 2.7% were double attachments (*N* = 30), and potentially one curve (0.09%) represented a triple attachment based on ddFLN4’s double-peaked mechanical fingerprint. The remaining curves exhibited noncanonical behavior and were not analyzed. For this assessment of the mechanical robustness of surface anchoring, force-extension curves with the correct contour length and a high mechanical rupture force (>1500 pN) that exhibited either no ddFLN4 unfolding events or a single-peak ddFLN4 event were interpreted as a single molecule, as these curves were most likely produced by a single, unfolded or partially unfolded ddFLN4 due to the brief time (∼200 ms) between adhesin-ligand rupture and successive pulls. However, as discussed below in the Data Analysis section, force-extension curves that did not exhibit the canonical double-peaked ddFLN4 unfolding mechanical fingerprint were not included in the final analysis. Given the total number of single molecule events at this specific location with ∼2 molecules, we conclude that individual single molecules were probed hundreds of times, verifying the surface covalent linkage was mechanically robust and not rupturing.

### Data Acquisition

We used a commercial AFM instrument (Cypher ES; Asylum Research) equipped with a temperature-controlled, closed fluidic-cell sample chamber. Collecting high-quality lifetime data required a large number of samples at each temperature and force, as error in exponential distribution sampling scales with $1/\sqrt{N}$ . Large *N* was also required for the constant-velocity data to yield appropriately asymmetric rupture force distributions and to achieve precise conversion of rupture force distributions to lifetimes as a function of force. To improve precision, we acquired each temperature data set with a single cantilever.^24^ To do so quickly, we implemented the ability to probe the same individual molecule multiple times, and to automatically find new molecules afterward. The large *N* and long lifetimes led to the data being acquired continuously over multiple days. On these time scales, the AFM detection laser drifts, requiring the sensitivity to be regularly and automatically updated. To assist in meeting these requirements, we developed an automated data-acquisition software (Cryptid, see Supporting Information for details).

For the constant-velocity assay in the standard pulling geometry (Figure 1B), the automated detection of a molecule being stretched used two criteria: (1) a rupture force exceeding 300 pN and (2) an extension at rupture between 30–150 nm. These conditions helped filter out surface adhesion, so the software did not spend time at locations where a molecule was not present. The force criteria were set loosely since we essentially never measured ruptures below 1,000 pN in the high-force pulling geometry (Figure S7B). After detection, the software repeatedly probed the same molecule as described in the Cryptid description in the Supporting Information.

For the constant-force assay, initial retraction was done at 1–2 μm/s until a force of 200 pN was reached. No extension filtering was used. After this trigger, a constant-force routine was initiated. This started a force-feedback loop that retracted the cantilever until the desired force was reached. This force was maintained until a rupture event, which led to rapid cantilever retraction as the feedback loop tried to maintain the set force. The hydrodynamic force (∼30 pN) generated by this high-speed retraction is visible in the bottom of Figure 3C and stopped after a preset limit to cantilever retraction was reached (∼2 μm). After rupture, the software brought the tip back to the surface and repeatedly stretched the same molecule again.

For SdrG:Fgβ, the constant-velocity data was digitized at a rate between 16–50 kHz, depending on velocity, and the constant-force data was digitized at 1 kHz. For SdrE:CFH, the constant-force data was digitized at 1 kHz, 2 kHz, 10 kHz, and 50 kHz for 0.8 nN, 1 nN, 1.2 nN, and 1.4 nN and over, respectively.

### Force Drift Rate Characterization

We used Olympus BioLever Mini cantilevers, which were calibrated in air using the thermal method^54^ (Table S1). Cantilevers were loaded and allowed to settle until the force drift dropped below 16 pN/min, typically after 3 h. As discussed above, in a constant-force assay using feedback, drift in the zero-force position of the cantilever^29^ leads to an unmeasured change in the applied force.^30^ Therefore, it is critical to quantify this drift. To do so, we collected cantilever position data for 5 min at 50 kHz at ∼300 nm off the surface. We then calculated the force precision as a function of the averaging time (i.e., the Allan deviation) (Figure S2, S8). At longer averaging times, the Allan deviation increases linearly. In this linear-drift regime, the drift rate can be approximated by $D=a\sqrt{2}$ , where *a* represents the slope of the force precision-vs-averaging time at long times. For the SdrG:Fgβ data set, the drift measurements with the steepest slope (10 °C) yielded a linear-drift rate of ∼16 pN/min. A dashed black line is included in Figure S2 to provide reference for this drift rate. Using this largest measured drift rate as our metric, almost all constant-force traces (>99.7%) experienced an estimated fractional force drift of <5%, a minor perturbation to the unbinding rate. The 5 longest traces included in the lifetime data (SdrG:Fgβ, 30 °C, 1000 pN) had fractional force drift between 5–7.3%. For the SdrE:CFH data, the individual cantilever used was particularly stable, with drift rates starting at 11 pN/min after a 3-h incubation prior to the experiment and then dropping to 4 pN/min after 24 h. Only the 800-pN traces were taken after 24 h. For the SdrE:CFH data included in the lifetime analysis (Figure 5B, 1000–1800 pN), >98.8% of curves had fractional force drift of <5%, with the longest traces (1000 pN) experiencing an estimated fractional force drift of 5–6.3%. For the 800-pN data, this fractional unmeasured change in force was <11%. We did not include the 800-pN data in the lifetime analysis due to the relatively large fractional drift due to the very long lifetimes. For a limited number of samples (*N* = 11), five traces exceeded 15 min.

### Data Analysis

Force curves were calculated by taking the measured vertical quad-photodiode voltage and multiplying by the measured inverse optical lever sensitivity (invOLS) and the cantilever spring constant (Table S1). Extension was calculated by subtracting the cantilever deflection from the measured stage position. For the physiologically pulling geometry (Figure 1B), both the constant-velocity and constant-force records contained ddFLN4 unfolding. Curves that did not show the characteristic double-peaked unfolding of ddFLN4,^21^ contained additional unfolding events, or had contour lengths substantially larger than expected (>106 nm) were not included in the analysis. The ddFLN4 had an average unfolding forces of 70.4 ± 0.7 pN (±SEM) and 80.7 ± 0.8 pN (*N* = 516) for the first and second unfolding events respectively at *v* = 3 μm/s.

Rupture forces and loading rates for the constant-velocity data (Figures 2, S5) were determined by fitting the last 1 nm prior to the rupture event to a line. The slope of this line with respect to time (∂*F*/∂*t* or *Ḟ*) is reported as the loading rate. The rupture force was taken to be the force where the data last crosses this fitted line. For the SdrE:Fgβ data, the probability distributions were generated by binning the measured rupture forces for each velocity (100, 300, 1000, 3000, and 10000 nm/s) with bin sizes of 31, 31, 34, 26, and 43 pN respectively. For the SdrE:CFH data, the probability distributions were generated by binning the measured rupture forces for each velocity (100, 500, 2500, and 10000 nm/s) with bin sizes of 73, 77, 83, and 71 pN, respectively.

Data in Figure 2 (left) was fit to a Bell–Evans model,^10,55^ while rupture force distributions (right) were fit to the linear-cubic DHS model for probability distributions,^19^ such that

5 $$p\left(F,Ḟ\right)={Ḟ}^{-1}k\left(F\right)e^{k_{B}Tk_{0}/\Delta x^{‡}Ḟ}\times e^{-k_{B}Tk\left(F\right)/\Delta x^{‡}Ḟ{\left[1-\left(\frac{2F\Delta x^{‡}}{3\Delta G^{‡}}\right)\right]}^{3/2}}$$

where *k*_0_ and *k*(*F*) are as reported in the main text (eq 2, 3). However, we note that fitting the rupture force distributions shown in Figure 2 to the DHS model resulted in a high correlation (0.93) between the attempt frequency (*A*) and Δ*G* ^‡^, indicating that these values were not independently constrained. Here, we used the fits to highlight the theoretically expected asymmetry of these distributions. Bins with *N* < 3 are not shown in Figure 2 (right panel) for clarity but were used in the fits to the DHS model.

Rupture force distributions were converted to lifetime measurements using a formula developed by Dudko, Hummer, and Szabo,^19^

6 $$k\left(F\right)=\frac{KVp\left(F|V\right)}{1-\int_{0}^{F}p\left(F^{\prime }|V\right)dF^{\prime }}$$

where *p*(*F|V*) is the rupture force probability distribution, *K* is the spring constant, and *V* is the retract velocity. We used the measured average loading rate for each distribution instead of the approximated loading rate *KV*. Bins with *N* < 3 in the rupture-force distributions were excluded to prevent skewing the data with under-sampled force ranges.

Lifetimes were found from constant force-data by measuring the difference between the rupture time and the time when the desired force was reached (typically ∼5 ms after initiation of the feedback loop). Both measurements were given a ±15 pN window to account for potential noise and Brownian fluctuations. For the SdrG:Fgβ assays, the maximum applied constant force for each temperature was set such that the lowest average lifetimes were >50 ms. This limitation was primarily due to the sampling rate of 1 kHz and the chosen analysis method of cumulative probability distributions. When compared to histograms, cumulative probability distributions have the advantage of using every data point individually, rather than relying on binning into arbitrary time bins. However, cumulative probability distributions can be skewed when the spacing between ordered lifetime measurements approaches the instrumental resolution (1 ms, in this case). Therefore, a cutoff of 20 ms was chosen such that lifetimes under the cutoff were not considered. This ensured that the spacing between sequential lifetime measurements was greater than the 1 ms time resolution for our shortest data sets, preventing artificially low time constants. Cumulative probability distributions were fit to exponential functions with an x-offset (time), which accounted for the 20 ms cutoff. Note that this limitation was not present in the SdrE:CFH data due to higher sampling rates, and therefore this cutoff was not required for that data set. Reported average lifetimes in the main text are this fitted time constant. Bootstrap analysis^31^ was done on each data set, generating 100,000 simulated data using sample with replacement. We used the standard deviation of this data as the estimated measurement error (Table S2). Using the SD reported by the fitting routine often led to unrealistically low values, well below what is estimated using simple counting statistics.

We performed a global fit the data presented in Figures 4B and 4C to the DHS model to determine Δ*x* ^‡^, Δ*H* ^‡^, Δ*S* ^‡^, and *A*. To sample a physically real parameter space, we imposed constraints on these values during the fitting, such that 0.03 nm ≤ Δ*x* ^‡^ ≤ 0.15 nm, 10 *k*_B_ *T* ≤ Δ*H* ^‡^ ≤ 100 *k*_B_ *T*, – 20 *k*_B_ *T* ≤ *T*Δ*S* ^‡^ ≤ 20 *k*_B_ *T*, and 3 ≤ log­(*A*) ≤ 14. To ensure that the fit was not stuck in a local minimum of χ^2^-space, each fit was repeated 1,000 times with different randomly sampled starting conditions over the range of allowed values. Two fits were done using this methodology: one where all variables were free (within the above constraints), and one where Δ*S* ^‡^ was fixed at 0. In the free fit, we determined Δ*x* ^‡^ = 0.046 ± 0.013 nm (fit ± SD) and Δ*H* ^‡^ = 40 ± 4 *k*_B_ *T*. These fitted values were essentially invariant over all 1000 starting conditions. Specifically, standard deviation of the 1000 fitted values was 0.0004 nm for Δ*x* ^‡^ and 0.16 *k*_B_ *T* for Δ*H* ^‡^, much less than the reported SD in the fitted value. In the constrained fit (Δ*S* ^‡^ = 0), we determined Δ*x* ^‡^ = 0.047 ± 0.0013, Δ*H* ^‡^ = 40 ± 1.6 *k*_B_ *T*, and log­(*A*) = 10.8 ± 0.7. Under this constraint, the standard deviations of the 1000 fitted values starting at different initial parameter values were even smaller (5 × 10^–8^ nm, 0.0005 *k*_B_ *T*, and 0.0002, respectively).

Table S3 compares the Δ*x* ^‡^ values estimated from the global DHS fit to data in Figure 4B,C and the Bell fits in Figure S3A. We see that the individual Δ*x* ^‡^ values obtained from the Bell fits are lower than those extracted from the global DHS fits. To test whether this was a result of the global fitting or from the differences in the models, we used the Bell model to fit a set of data generated from the DHS model using the parameters determined from fitting Figure 4B,C. We see that the Bell model analysis of the DHS data also yielded a lower Δ*x* ^‡^ (0.039–0.040 nm vs 0.046 nm, respectively). We note that these values derived from the Bell fitting to the synthetic DHS data were in relative agreement with the Bell fits the individual data sets. This suggests that small yet systematically lower Δ*x* ^‡^ for the Bell fits were a result of differences between the two models.

### Poisson Analysis for Estimating Pulling on Single versus Multiple Molecules

To estimate the frequency of multiple attachments using a specified tip-ligand attachment rates, we used a Poisson analysis.^38^ We approximate the count of multiple attachments using Poisson counting statistics,

7 $$P\left(k\right)=\frac{\lambda^{k}e^{-\lambda }}{k!}$$

where *P* is the probability of measuring *k* events given an average number of events λ. The single-cell force spectroscopy of SdrE:FH reports an attachment frequency of 47%,^18^ giving *P*(0) = 0.53 and therefore λ = 0.63. Given this, the probability of measuring a multimer (*k* ≥ 2) is *P*(*k* ≥ 2) = 1 – *P*(0) – *P*(1) = 0.13. As *P*(1) = 0.34, we estimate 28% of all attachments to be multimers given the tip attachment frequency of 47%.

## Supplementary Material



## Data Availability

The data that supports the findings of this paper are openly available on Dryad (doi: 10.5061/dryad.tmpg4f5fx).

## Notes

Details on materials and methods, including a description of the data-acquisition software, the polyprotein constructs; figures showing the surface-attachment chemistry, Allan deviation, the temperature dependence of Δ*x* ^‡^, covariance of log­(*A*) and *T*Δ*S* ^‡^, the SdrE:CFH rupture force data and lifetimes; and tables summarizing the cantilever properties and reported lifetimes (PDF)
