# Supplementary material for: The Temperature Dependence of the Pathogenic Bacterial Adhesin Complex SdrG:Fgβ under Sustained Loads: A High-Force Slip Bond
Source: ACS Nano. 2026 Oct 23;20(44):29620–32. doi: 10.1021/acsnano.6c10566 (PMC13758387; doi:10.1021/acsnano.6c10566)
Supplement: Supplementary file 1 [file nn6c10566_si_001.pdf]

# Supporting Information for

# The Temperature Dependence of the Pathogenic Bacterial Adhesin Complex SdrG:Fgβ under Sustained Loads: a High-Force Slip Bond

Brandon Dzuba,[1] Surya Pratap S. Deopa,[1] Marc-André LeBlanc,[1] Lukas F. Milles,[2,3] and Thomas T. Perkins[1,4,*]

[1]JILA, National Institute of Standards and Technology and University of Colorado, Boulder, CO 80309;

[2]Gene Center, LMU Munich, Feodor-Lynen-Str. 25, 81377 Munich, Germany;

[3]Max Planck Institute of Biochemistry, Am Klopferspitz 18, 82152 Martinsried, Germany;

[4]Department of Molecular, Cellular, and Developmental Biology, University of Colorado, Boulder, CO 80309

*Corresponding email: tperkins@colorado.edu

**Materials and Methods** S2–3

**Polyprotein sequences** S3–4

**Figures:**
Figure S1 S4
Figure S2 S5
Figure S3 S5
Figure S4 S6
Figure S5 S6
Figure S6 S7
Figure S7 S7
Figure S8 S8

**Tables:**
Table S1 S9
Table S2 S10
Table S3 S11

**References** S12

## Materials and Methods

**Automated data-acquisition software.** Cryptid is a general-purpose AFM single-molecule force spectroscopy (SMFS) software designed for Asylum Research Cyphers and built inside Asylum Research's AR16+ software (Igor Pro 6). To probe the surface, Cryptid has a built-in grid system with a user defined spacing. When a molecule is found, Cryptid can stretch that molecule repeatedly until a user-defined number of successive attempts fail. If desired, a finer-spaced sub-grid is searched in the region to re-find the molecule. At each point, a user-defined series of tasks (modules) can be performed once a molecule is found. Currently, several useful modules include constant-velocity assays, constant-force assays, tip–surface-attachment centering (DNA or polyprotein),[1] zig-zag ramps,[2] and equilibrium assays.[3] Modules designed to facilitate in long-term data acquisition are also included, such as automated photodiode zeroing and automated sensitivity updating through collection and fitting of a power spectral density (PSD) measurements. Each module can be executed successively in any order or individually, facilitating the design of custom complex assays that can be saved for reuse. Lastly, users can write their own modules, expanding the software with additional functionality. Software can be downloaded at https://github.com/perkinslab/cryptid.

For both constant-velocity and constant-force assays, Cryptid's grid was set to a 50 nm-spaced x-y grid. Once a molecule was found, we required 20–30 successive failed attachments before moving onto the next spot. A finer, 3-nm-spaced 3×3 grid was used to re-find molecules after detachment. At each spot, the cantilever was pressed into the surface (50–100 pN) for ~10 ms, followed by retraction at the set retraction velocity. If a molecule was detected (criteria below, dependent on experiment type), repeated measurements were made until the molecule was lost, indicated by no additional attachments. After every 100–200 force curves, the cantilever retracted to ~300 nm from the surface and the sensitivity was updated. This was done using the Cryptid module described above, where a PSD was collected and the $0^{th}$ order peak was fit to a simple harmonic oscillator model, extracting $\langle x^2 \rangle$. The equipartition theorem was then applied with the

known spring constant and the measured <$x^2$> to determine the sensitivity. Such updates typically resulted in <2% changes in the sensitivity, with no systematic changes throughout the experiments.

**Polyprotein sequences:**

**SdrG:** SdrG(N2,N3)-his-ybbR

MGTEQGSNVNHLIKVTDQSITEGYDDSDGIIKAHDAENLIYDVTFEVDDKVKSGDTMTV
NIDKNTVPSDLTDSFAIPKIKDNSGEIIATGTYDNTNKQITYTFTDYVDKYENIKAHLKLTS
YIDKSKVPNNNTKLDVEYKTALSSVNKTITVEYQKPNENRTANLQSMFTNIDTKNHTVE
QTIYINPLRYSAKETNVNISGNGDEGSTIIDDSTIIKVYKVGDNQNLPDSNRIYDYSEYED
VTNDDYAQLGNNNDVNINFGNIDSPYIIKVISKYDPNKDDYTTIQQTVTMQTTINEYTGE
FRTASYDNTIAFSTSSGQGQGDLPPEKTELKLPRSRHHHHHHGSLEVLFQGPDSLEFIASK
LA

**SdrE:** SdrE(N2,N3)-his-ybbR-sortase

MATAVAQPAAVASNNVNDLIKVTKQTIKVGDGKDNVAAAHDGKDIEYDTEFTIDNKVKK
GDTMTINYDKNVIPSDLTDKNDPIDITDPSGEVIAKGTFDKATKQITYTFTDYVDKYEDIK
SRLTLYSYIDKKTVPNETSLNLTFATAGKETSQNVTVDYQDPMVHGDSNIQSIFTKLDED
KQTIEQQIYVNPLKKSATNTKVDIAGSQVDDYGNIKLGNGSTIIDQNTEIKVYKVNSDQQ
LPQSNRIYDFSQYEDVTSQFDNKKSFSNNVATLDFGDINSAYIIKVVSKYTPTSDGELDIA
QGTSMRTTDKYGYYNYAGYSNFIVTSNDTGGGDGTVKPEEKSGHHHHHHGSDSLEFIAS
KLASLPETGG

**Fgβ:** Fgβ-ddFLN4-his-ybbR

MGTNEEGFFSARGHRPLDGSGSGSGSAGTGSGADPEKSYAEGPGLDGGESFQPSKFKIH
AVDPDGVHRTDGGDGFVVTIEGPAPVDPVMVDNGDGTYDVEFEPKEAGDYVINLTLDG
DNVNGFPKTVTVKPAPSGHHHHHHGSDSLEFIASKLA

**CFH:** CFH-ddFLN4-his-ybbR

MATRLSSRSHTLRTTCWDGKLEYPGSGSGSGSAGTGSGADPEKSYAEGPGLDGGESFQP
SKFKIHAVDPDGVHRTDGGDGFVVTIEGPAPVDPVMVDNGDGTYDVEFEPKEAGDYVIN
LTLDGDNVNGFPKTVTVKPAPSGHHHHHHGSDSLEFIASKLA

**CFH (reverse):** ybbR-his-$\alpha_3$D-CFH

MDSLEFIASKLAHHHHHHGSGSGSGSMGSWAEFKQRLAAIKTRLQALGGSEAELAAFE
KEIAAFESELQAYKGKGNPEVEALRKEAAAIRDELQAYRHNGSGSGSGSAGTGSGTRLS
SRSHTLRTTCWDGKLEYP

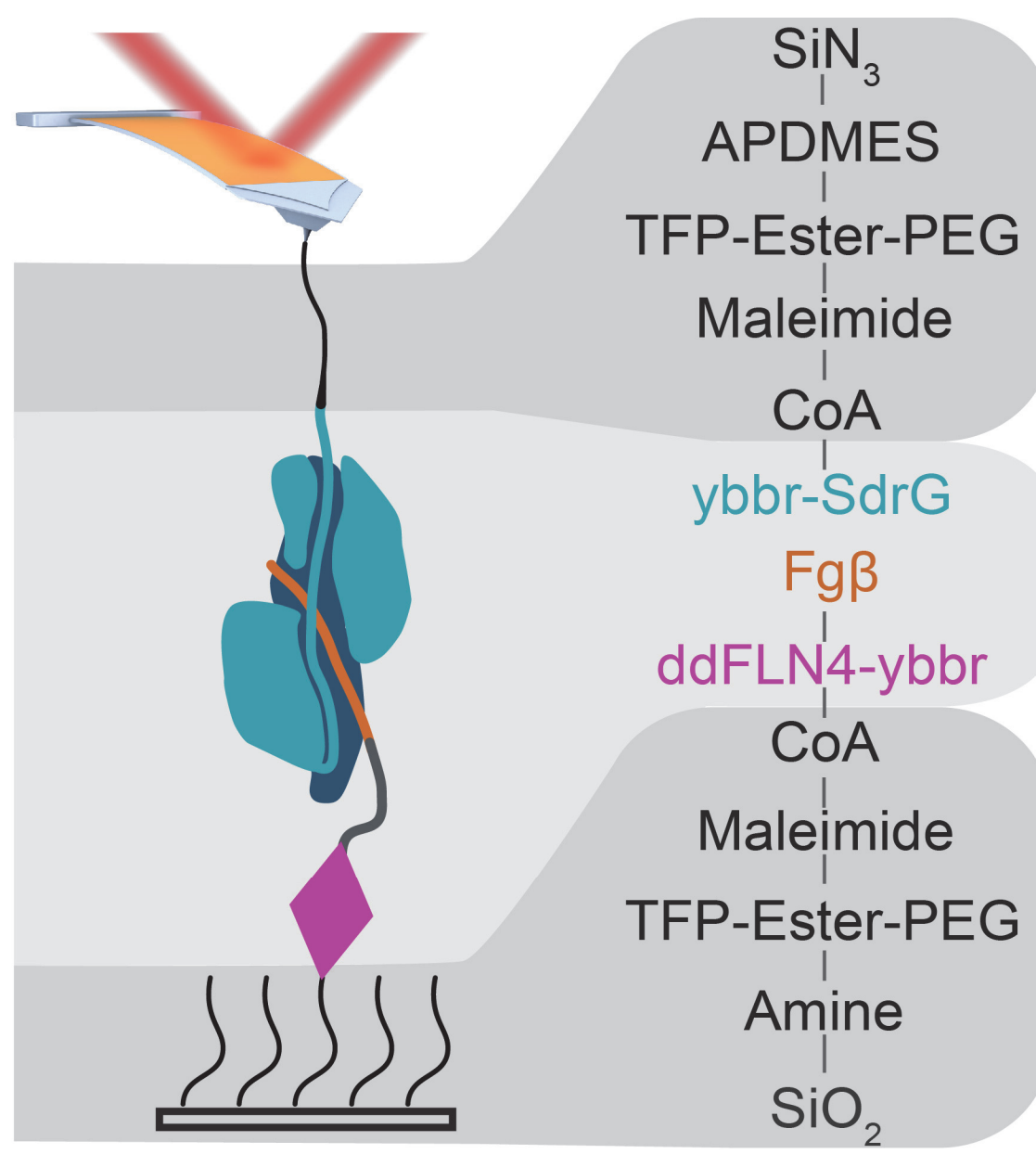


**Figure S1.** An illustration of the site-specific, covalent attachment scheme used in the single-molecule assay. The AFM cantilever was $NH_3$-functionalized using 3-aminopropoldimethylethoxysilane (APDMES). Both the $NH_3$-coated AFM tip and commercial $NH_3$-coated glass coverslips were PEG-coated using a TFP-ester–$PEG_{12}$–maleimide.[4] The maleimide was reacted with CoA, which contains a terminal thiol group. The protein constructs (*e.g.*, Fgβ-ddFLN4-ybbR and SdrG-ybbR) were each covalently anchored to the CoA-surfaces using the Sfp, which enzymatically conjugated the ybbR (a short genetic tag[5]) to CoA.

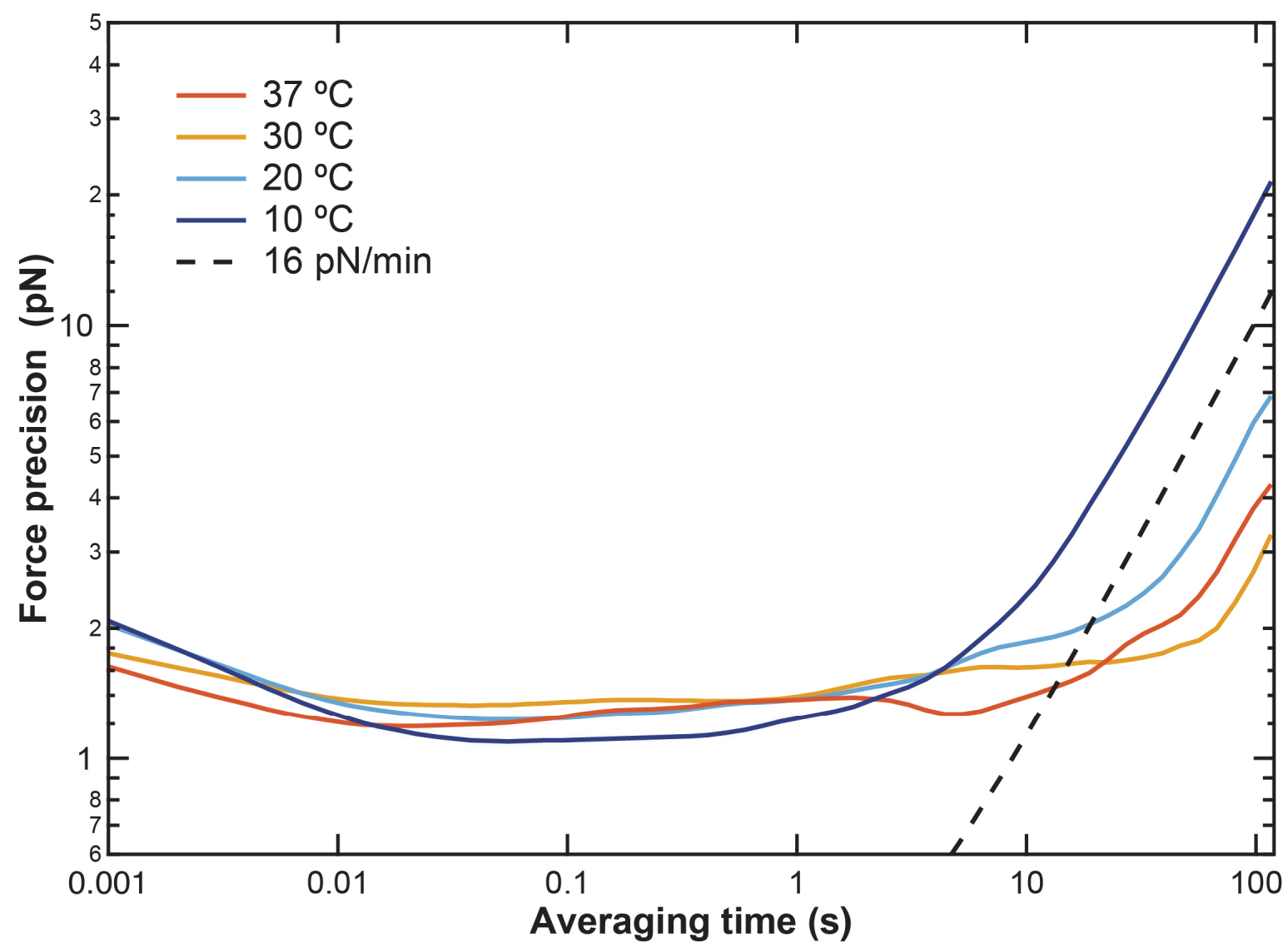


**Figure S2.** Force-precision versus averaging time (*i.e.*, Allan deviation) plotted at each temperature: 10 °C (dark blue), 20 °C (light blue), 30 °C (gold), and 37 °C (red) taken >3 h after loading. For comparison, the dashed line shows the slope corresponding that would result from a 16 pN/min linear force drift.

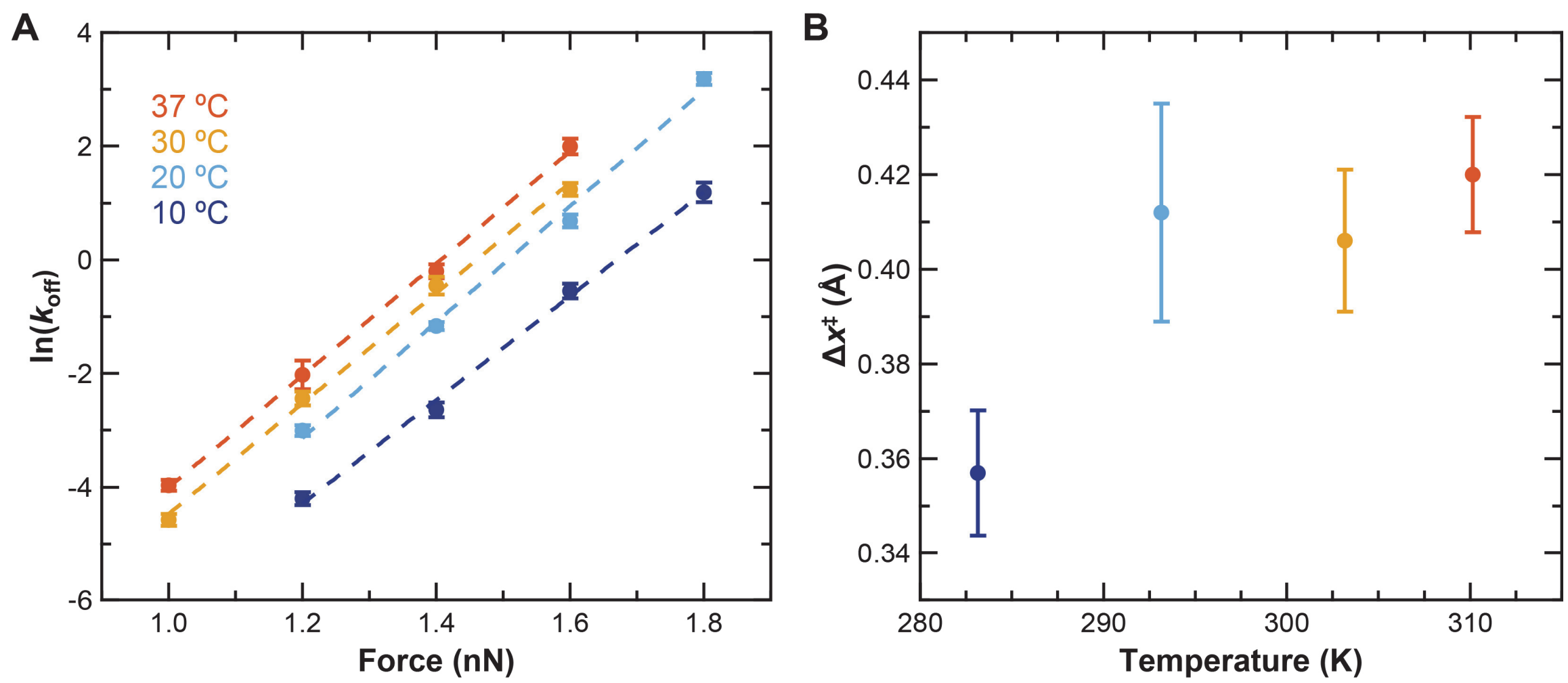


**Figure S3.** Bell model[6] fits to the SdrG:Fgβ constant-force lifetime data yield the distance to the transition state ($\Delta x^{\ddagger}$). (**A**) Natural logarithm of the off rate ($k_{off} = 1/\tau_{fit}$) plotted as a function of force across a range of temperatures: 10 °C (dark blue), 20 °C (light blue), 30 °C (gold), and 37 °C (orange), where the dashed lines represent Bell model fits to the individual temperature data sets. (**B**) The deduced distance to the transition state ($\Delta x^{\ddagger}$) plotted as a function temperature [0.036 ± 0.0013 nm (10 °C), 0.041 ± 0.0023 nm (20 °C), 0.041 ± 0.0015 nm (30 °C), and 0.042 ± 0.0012 nm (37 °C), where values represent the fit ± SD and the SD is the square root of the diagonal of the covariance matrix.

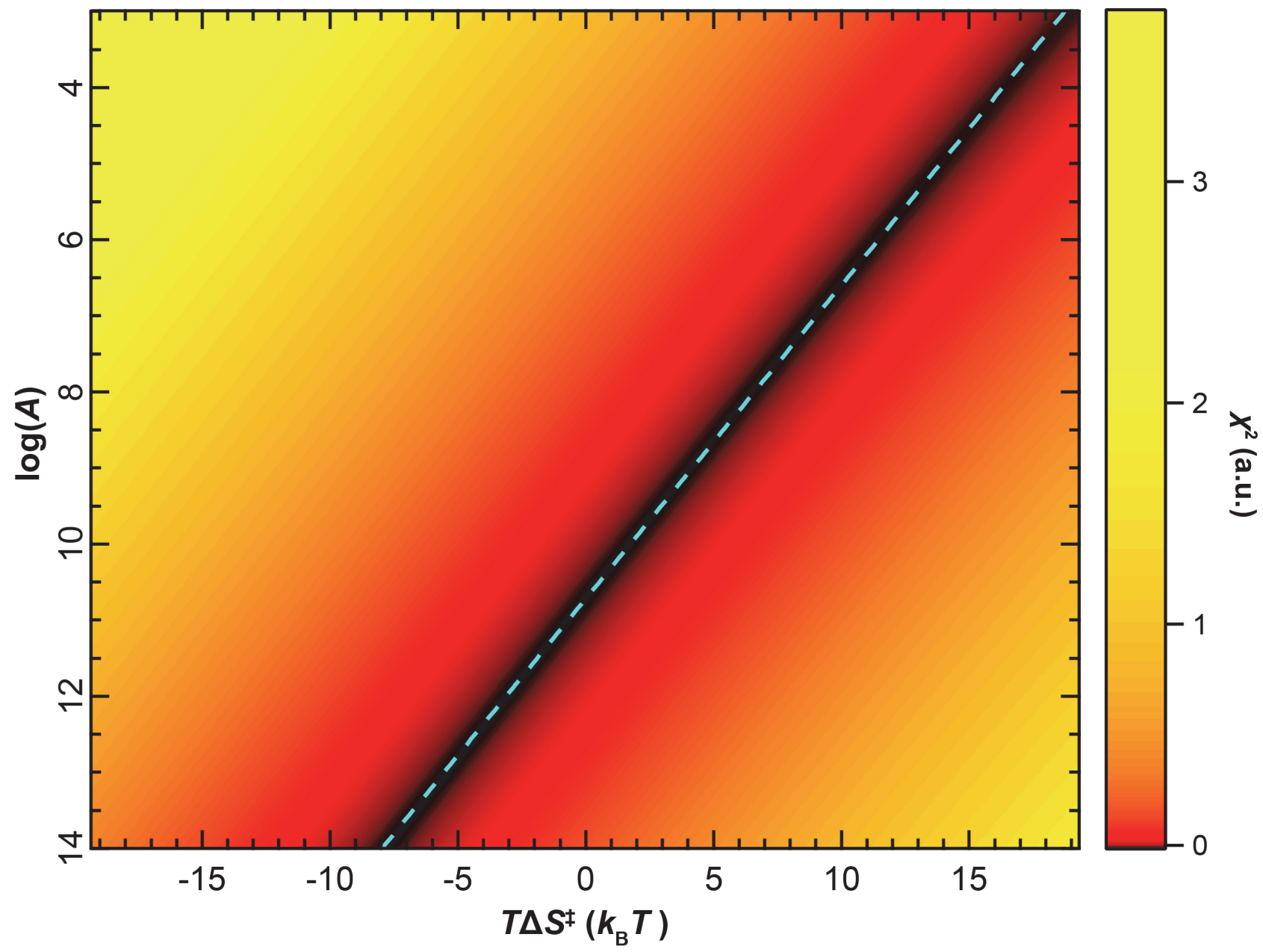


**Figure S4.** A 2-dimension heat map shows $\chi^2$ as a function of log ($A$) and $T\Delta S^\ddagger$ for the global fit to the data shown in Figure 4 B,C where the two other parameters in the DHS model were held constant ($\Delta x^\ddagger$ = 0.46 Å and $\Delta H^\ddagger$ = 40 $k_BT$). The dark region shows a band that represents a minimum of $\chi^2$, where the dashed line is described by $T\Delta S^\ddagger \approx 26 - 2.4\times\log(A)$.

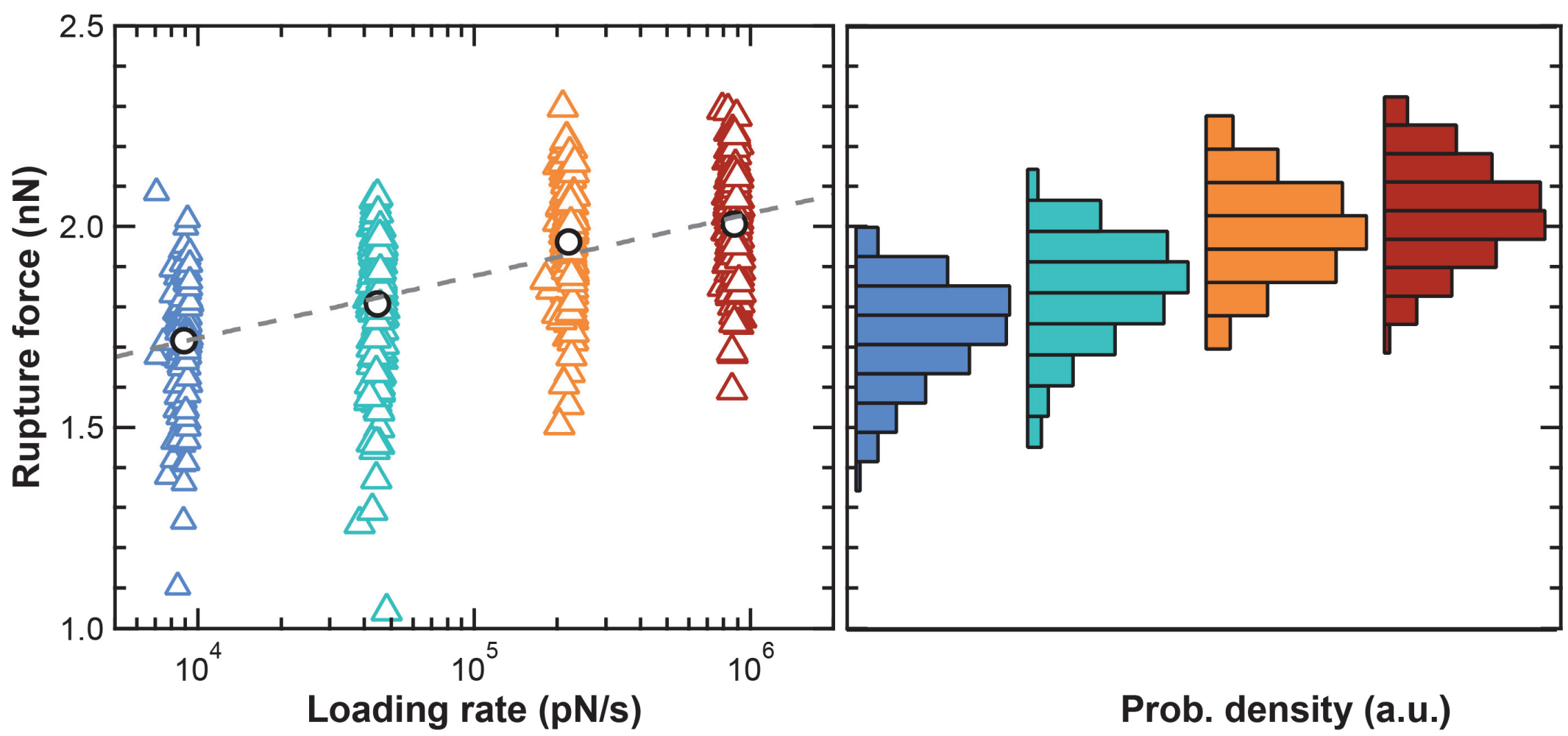


**Figure S5.** Rupture force of SdrE:CFH probed over a range of loading rates ($\partial F/\partial t$) in a constant-velocity assay. (Left) Triangles represent individual rupture force measurements, while circles represent the average for each loading rate. Different velocities represented by different colors: 100 nm/s (8,900 pN/s, blue), 500 nm/s (44,700 pN/s, teal), 2.5 µm/s (220,000 pN/s, orange) and 10 µm/s (874,000 pN/s, red). A Bell-Evans[7,8] fit to the average rupture force plotted in grey and yielded $\Delta x^\ddagger$ = 0.061 ± 0.007 nm (fit ± SD) and $\ln(k_0)$= −8.9 ± 1.4. (Right) Rupture force distributions plotted for the different loading rates ($N$ = 196, 210, 191, and 174, left to right respectively).

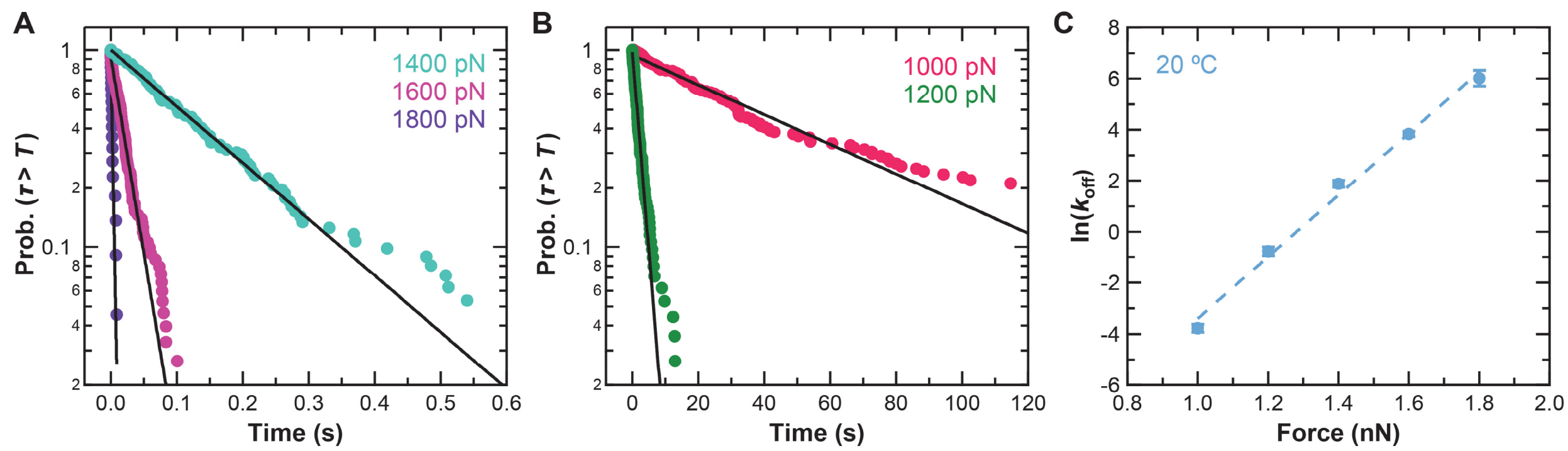


**Figure S6.** Kinetic analysis of SdrE:CFH constant-force data. (**A, B**) Logarithmic plots of the normalized cumulative probability distributions measured at 20 °C for 1800 pN (purple, $N = 22$, $\tau_{\text{fit}} = 0.0024 \pm 0.0006$), 1600 pN (pink, $N = 151$, $\tau = 0.0218 \pm 0.002$ s), 1400 pN (turquoise, $N = 112$, $\tau_{\text{fit}} = 0.15 \pm 0.02$ s), 1200 pN (green, $N = 113$, $\tau_{\text{fit}} = 2.3 \pm 0.3$ s), and 1000 pN (magenta, $N = 128$, $\tau_{\text{fit}} = 44 \pm 7$ s) from left to right respectively. (**C**) Natural logarithm of the off rate ($k_{\text{off}} = 1/\tau_{\text{fit}}$) plotted as a function of force. The dashed line represents a fit to the Bell-Evans model, yielding $\Delta x^{\ddagger} = 0.049 \pm 0.003$ nm and $\log(k_{\text{off}}) = -6.73 \pm 0.38$, where values represent the fit ± SD. Error bars in panel C were calculated by bootstrap analysis,[9] but in some cases are smaller than the symbol size.

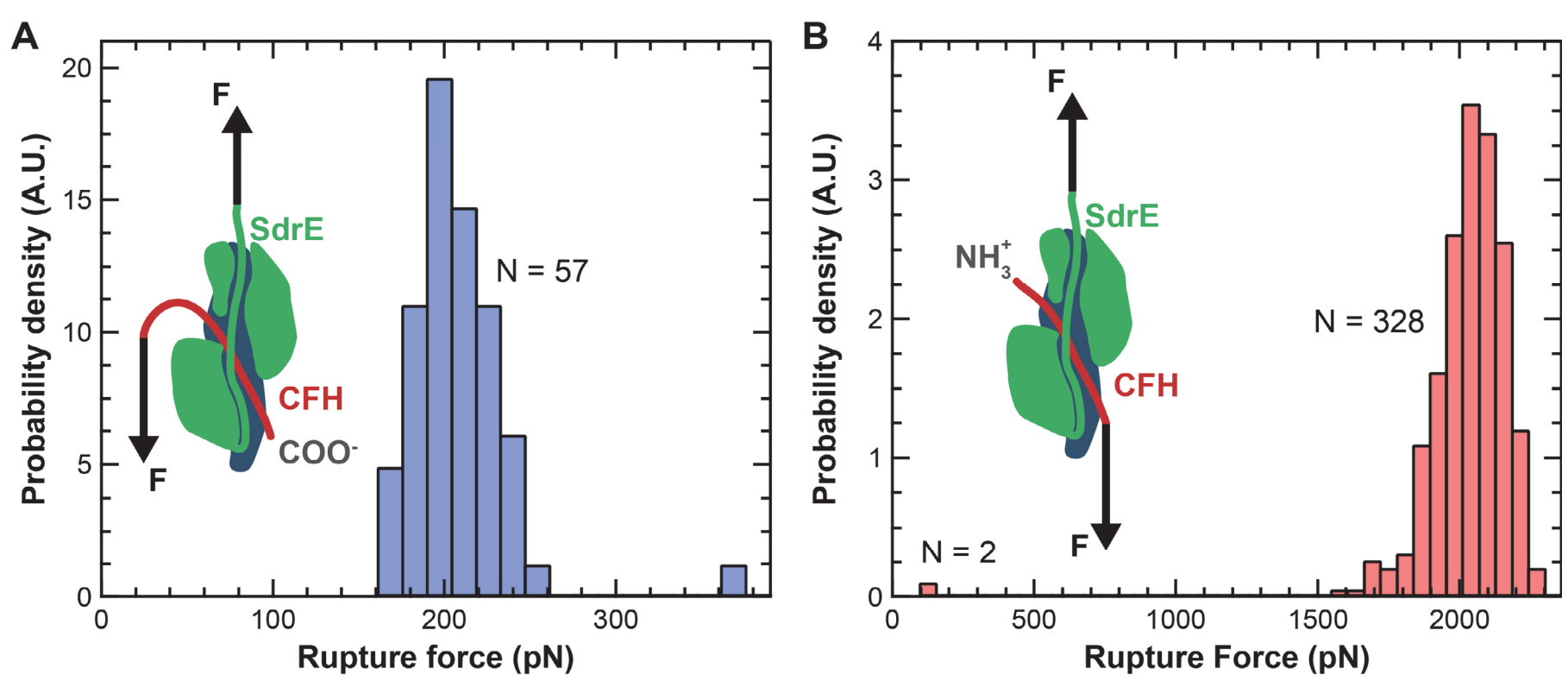


**Figure S7.** Rupture force varies with pulling geometry for SrdE:CFH. Rupture force probability distributions ($v = 1$ μm/s) for SdrE:CFH taken using **(A)** the reverse pulling geometry and **(B)** the standard pulling geometry. Insets are illustrations of the reverse and standard pulling geometry. In the reverse, non-canonical pulling geometry, force is applied to the N-terminal end of the CFH ligand. In the standard pulling geometry, force is applied to the C-terminal end of CFH. For the standard pulling geometry data set, we did not use an *in situ* force filter of 300 pN as described in Methods. Rather, all traces were recorded, and we analyzed all single-molecule records consistent with our described criteria (*e.g.*, correct contour length and ddFNL4 marker protein). This plot shows that we observed essentially no low-force ruptures in the standard pulling geometry at $v = 1$ μm/s. For the reverse pulling dataset, we used an *in situ* force filter of 30 pN to prevent the collection of traces without molecular attachment.

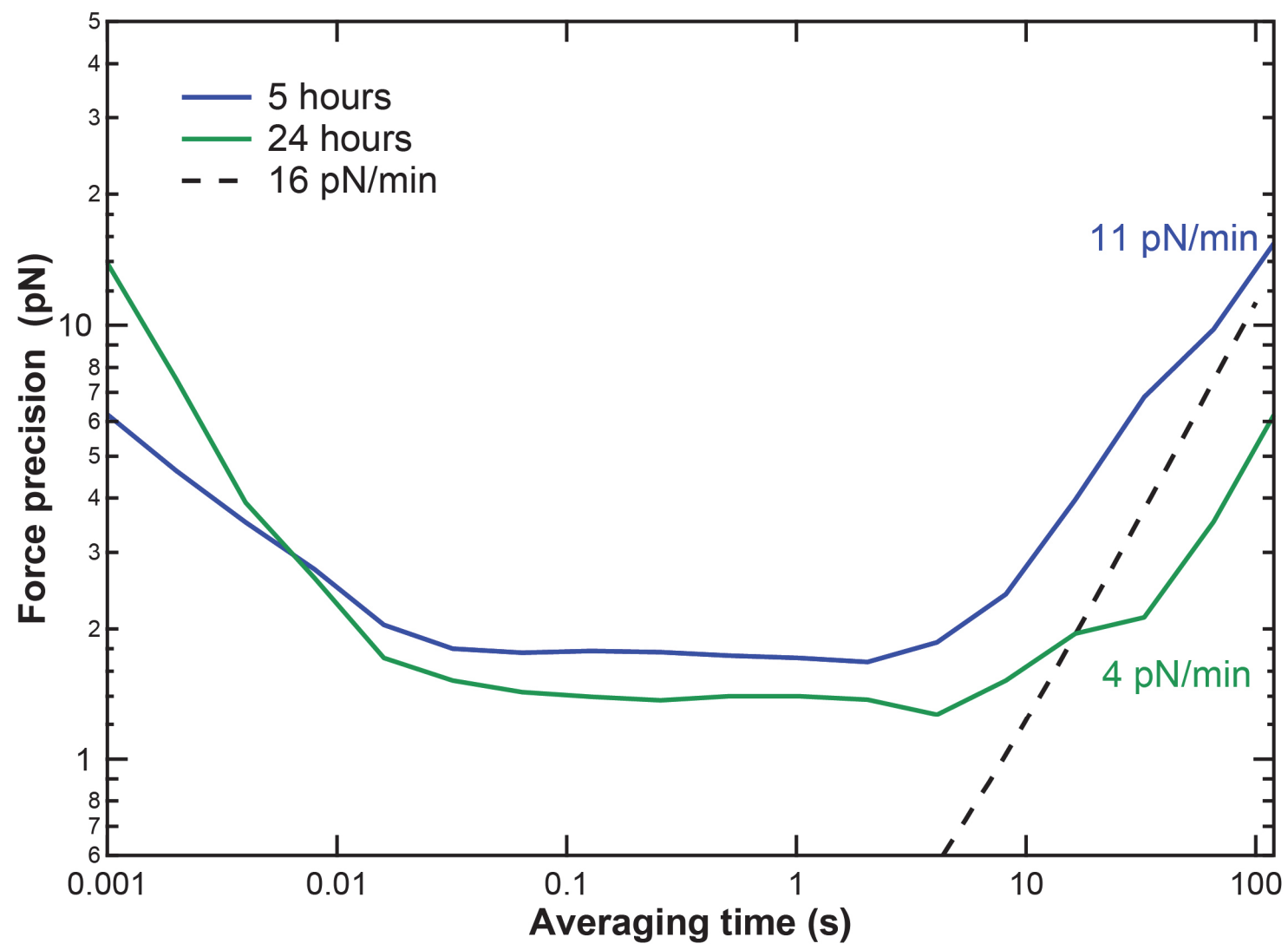


**Figure S8.** Force-precision versus averaging time (*i.e.*, Allan deviation) plotted for the cantilever used to acquire the SdrE:CFH data shown in Figure 5 at 5 h after loading (blue, 11 pN/min) and 24 h after loading (green, 4 pN/min). For comparison, the dashed line shows the slope corresponding that would result from a 16 pN/min linear force drift.

**Table S1. Cantilever and spring constants for each data set**

| Cantilever | Spring constant | Data set |
|---|---|---|
| M1 (BioLever Mini) | 141 pN/nm | 10 °C |
| M2 (BioLever Mini) | 140 pN/nm | 20 °C |
| M3 (BioLever Mini) | 129 pN/nm | 30 °C, 37 °C |
| M4 (BioLever Mini) | 116 pN/nm | SdrE:CFH, 20 °C |
| M5 (BioLever Mini) | 144 pN/nm | SdrE:CFH, No filter |
| M6 (BioLever Mini) | 145 pN/nm | SdrE:CFH, Reverse pulling |

**Table S2. Lifetime data**

| Experiment | Force (pN) | Lifetime fit (s) | Lifetime avg (s) | Bootstrap Error (± s) | Relative Error | *N* |
|---|---|---|---|---|---|---|
| SdrG: 10 °C | 1200 | 67.1 | 68.4 | 7.3 | 0.11 | 87 |
| SdrG: 10 °C | 1400 | 14.0 | 15.0 | 1.8 | 0.13 | 115 |
| SdrG: 10 °C | 1600 | 1.73 | 1.81 | 0.23 | 0.13 | 104 |
| SdrG: 10 °C | 1800 | 0.305 | 0.313 | 0.053 | 0.17 | 64 |
| SdrG: 20 °C | 1200 | 20.2 | 20.1 | 2.0 | 0.098 | 127 |
| SdrG: 20 °C | 1400 | 3.21 | 3.22 | 0.23 | 0.073 | 165 |
| SdrG: 20 °C | 1600 | 0.508 | 0.557 | 0.054 | 0.11 | 136 |
| SdrG: 20 °C | 1800 | 0.0415 | 0.0566 | 0.0043 | 0.10 | 66 |
| SdrG: 30 °C | 1000 | 97.4 | 83.1 | 9.9 | 0.10 | 49 |
| SdrG: 30 °C | 1200 | 11.5 | 10.5 | 1.4 | 0.12 | 61 |
| SdrG: 30 °C | 1400 | 1.57 | 1.73 | 0.25 | 0.16 | 86 |
| SdrG: 30 °C | 1600 | 0.290 | 0.29 | 0.033 | 0.11 | 65 |
| SdrG: 37 °C | 1000 | 53.0 | 48.7 | 5.0 | 0.095 | 80 |
| SdrG: 37 °C | 1200 | 7.5 | 8.7 | 1.9 | 0.25 | 79 |
| SdrG: 37 °C | 1400 | 1.22 | 1.15 | 0.15 | 0.12 | 78 |
| SdrG: 37 °C | 1600 | 0.137 | 0.170 | 0.019 | 0.14 | 75 |
| SdrE: 20 °C | 1000 | 43.7 | 64.4 | 6.8 | 0.16 | 128 |
| SdrE: 20 °C | 1200 | 2.15 | 2.52 | 0.33 | 0.17 | 113 |
| SdrE: 20 °C | 1400 | 0.152 | 0.167 | 0.019 | 0.14 | 112 |
| SdrE: 20 °C | 1600 | 0.0218 | 0.0213 | 0.0021 | 0.10 | 151 |
| SdrE: 20 °C | 1800 | 0.00242 | 0.00231 | 0.00060 | 0.31 | 22 |

**Table S3. Comparison of $\Delta x^‡$ for DHS and Bell-Evans models**

| Temperature (°C) | Global DHS $\Delta x^‡$ (nm) | Bell-Evans $\Delta x^‡$ (nm) | Bell-Evans fit to global DHS fit $\Delta x^‡$ (nm) |
|---|---|---|---|
| 10 | 0.046 ± 0.013 | 0.036 ± 0.0013 | 0.039 ± 0.000028 |
| 20 | 0.046 ± 0.013 | 0.041 ± 0.0023 | 0.039 ± 0.000028 |
| 30 | 0.046 ± 0.013 | 0.041 ± 0.0015 | 0.040 ± 0.000028 |
| 37 | 0.046 ± 0.013 | 0.042 ± 0.0012 | 0.040 ± 0.000028 |

**REFERENCES**

(1) Walder, R.; Van Patten, W. J.; Adhikari, A.; Perkins, T. T., Going Vertical to Improve the Accuracy in Atomic Force Based Single-Molecule Force Spectroscopy *ACS Nano* **2018**, *12*, 198−207.
(2) Jacobson, D. R.; Uyetake, L.; Perkins, T. T., Membrane-Protein Unfolding Intermediates Detected with Enhanced Precision Using a Zigzag Force Ramp. *Biophys. J.* **2020**, *118*, 667–675.
(3) Edwards, D. T.; LeBlanc, M. A.; Perkins, T. T., Modulation of a Protein-Folding Landscape Revealed by AFM-Based Force Spectroscopy Notwithstanding Instrumental Limitations. *Proc. Natl. Acad. Sci. U.S.A.* **2021**, *118*.
(4) Deopa, S. P. S.; Bjorkman, K. K.; Edwards, D. T.; LeBlanc, M. A.; Buvoli, M.; Oakes, S. H.; Dzuba, B.; Karabina, A.; Leinwand, L. A.; Perkins, T. T., Force-Induced Ankle Opening Reveals Mechanical Stabilization of the Ankle of Human β-Cardiac Myosin. *ACS Nano* **2026**.
(5) Yin, J.; Straight, P. D.; McLoughlin, S. M.; Zhou, Z.; Lin, A. J.; Golan, D. E.; Kelleher, N. L.; Kolter, R.; Walsh, C. T., Genetically Encoded Short Peptide Tag for Versatile Protein Labeling by Sfp Phosphopantetheinyl Transferase. *Proc. Natl. Acad. Sci. U.S.A.* **2005**, *102*, 15815–15820.
(6) Bell, G. I., Models for Specific Adhesion of Cells to Cells. *Science* **1978**, *200*, 618–627.
(7) Evans, E.; Ritchie, K., Dynamic Strength of Molecular Adhesion Bonds. *Biophys. J.* **1997**, *72*, 1541–1555.
(8) Evans, E.; Ritchie, K., Strength of a Weak Bond Connecting Flexible Polymer Chains. *Biophys. J.* **1999**, *76*, 2439–2447.
(9) Efron, B.; Tibshirani, R., *An Introduction to the Bootstrap*. Chapman and Hall: New York, 1993.
